# Menke-Hennekam syndrome; delineation of domain-specific subtypes with distinct clinical and DNA methylation profiles

**DOI:** 10.1016/j.xhgg.2024.100337

**Published:** 2024-09-21

**Authors:** Sadegheh Haghshenas, Hidde J. Bout, Josephine M. Schijns, Michael A. Levy, Jennifer Kerkhof, Pratibha Bhai, Haley McConkey, Zandra A. Jenkins, Ella M. Williams, Benjamin J. Halliday, Sylvia A. Huisman, Peter Lauffer, Vivian de Waard, Laura Witteveen, Siddharth Banka, Angela F. Brady, Elena Galazzi, Julien van Gils, Anna C.E. Hurst, Frank J. Kaiser, Didier Lacombe, Antonio F. Martinez-Monseny, Patricia Fergelot, Fabíola P. Monteiro, Ilaria Parenti, Luca Persani, Fernando Santos-Simarro, Brittany N. Simpson, MKHK Research Consortium, Mariëlle Alders, Stephen P. Robertson, Bekim Sadikovic, Leonie A. Menke

## Main text

(Human Genetics and Genomics Advances *5*, 100287; July 18, 2024)

The following corrections have been made since publication of this manuscript: an additional reference number for the Amsterdam UMC medical ethics committee was added, and an additional row for “cerebral anomalies” was added to tables 1 and 2. Additionally, the supplemental files were incorrectly named, and this has now been fixed. The authors regret the errors.

