# Supplementary material for: Menke-Hennekam syndrome; delineation of domain-specific subtypes with distinct clinical and DNA methylation profiles
Source: HGG Adv. 2024 Mar 29;5(3):100287. doi: 10.1016/j.xhgg.2024.100287 (PMC11040166; doi:10.1016/j.xhgg.2024.100287)
Supplement: Document S1. Figure S1, Table S1, S4, and S5 [file mmc1.pdf]

HGGA, Volume *5*

Supplemental information

Menke-Hennekam syndrome; delineation of domain-specific subtypes with distinct clinical and DNA methylation profiles

Sadegheh Haghshenas, Hidde J. Bout, Josephine M. Schijns, Michael A. Levy, Jennifer Kerkhof, Pratibha Bhai, Haley McConkey, Zandra A. Jenkins, Ella M. Williams, Benjamin J. Halliday, Sylvia A. Huisman, Peter Lauffer, Vivian de Waard, Laura Witteveen, Siddharth Banka, Angela F. Brady, Elena Galazzi, Julien van Gils, Anna C.E. Hurst, Frank J. Kaiser, Didier Lacombe, Antonio F. Martinez-Monseny, Patricia Fergelot, Fabíola P. Monteiro, Ilaria Parenti, Luca Persani, Fernando Santos-Simarro, Brittany N. Simpson, MKHK Research Consortium, Mariëlle Alders, Stephen P. Robertson, Bekim Sadikovic, and Leonie A. Menke

Table of contents

Figure S1. Lateral facial and distal limb morphology of the presently described individuals with a variant in the MKHK region of *CREBBP* and *EP300*. 2

Table S1. Summary of the 3-step process for the selection of probes 8

Table S2. Morphological characteristics of individuals with a confirmed MKHK methylation profile or episignature (in ID4) per MKHK subtype. 8

Table S3 (see excel file). Clinical characteristics and morphological features of the presently described individuals with a variant in the MKHK region of *CREBBP* and *EP300*. 9

Table S4 (see excel file). Genotypes, episignatures and in silico prediction scores of all presently described individuals with a variant in the MKHK region of CREBBP (NM_004380.2) or EP300 (NM_001429.3). 9

Table S5. List of identified differentially methylated regions. 10

References 12

Figure S1. Lateral facial and distal limb morphology of the presently described individuals with a variant in the MKHK region of *CREBBP* and *EP300*.

The lateral facial characteristics, the hands and feet of individuals with a variant in (A) the ZZ-domain, (B) TAZ2-domain, (C) ID4, and (D) in between the ZZ and TAZ2-domain (C.ZT.1 en C.ZT.2) and in between TAZ2 and ID4 (C.TI.29). Study numbers are depicted within each photograph reflecting the affected gene (C or E representing *CREBBP* and *EP300*) and domain (Z, T, I, ZT, and TI representing ZZ, TAZ2, ID4, the region between ZZ and TAZ2, and the region between TAZ2 and ID4, respectively) followed by a unique number. The study numbers of individuals with a confirmed MKHK episignature or methylation profile are displayed in bold, and of individuals who were tested, but in whom no MKHK episignature or methylation profile was found, in italics. Detailed description of facial and distal limb morphology of all individuals can be found in Table S3.

A

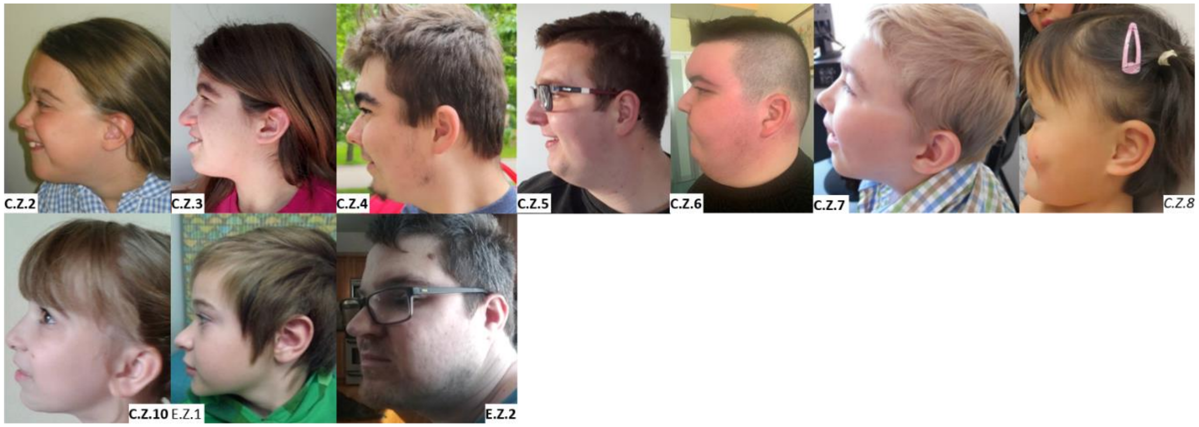

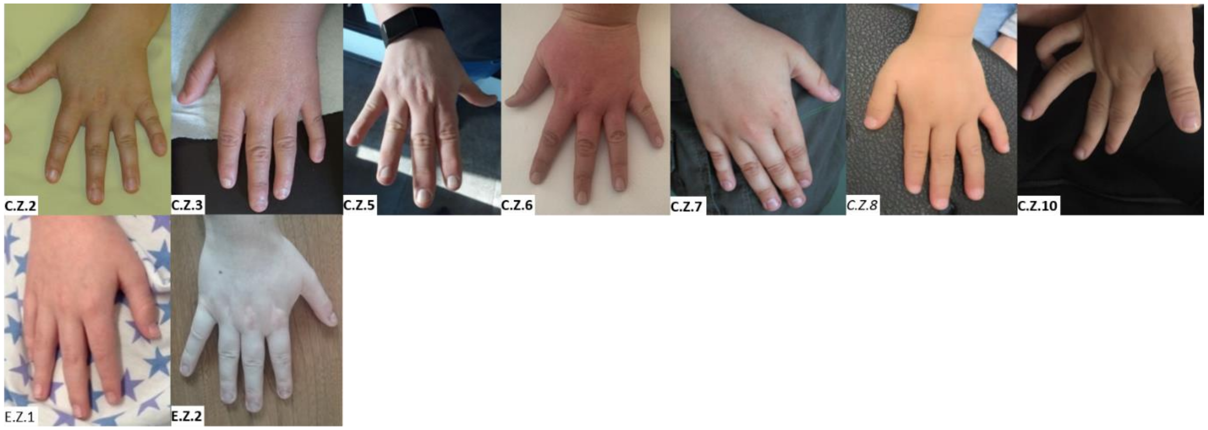

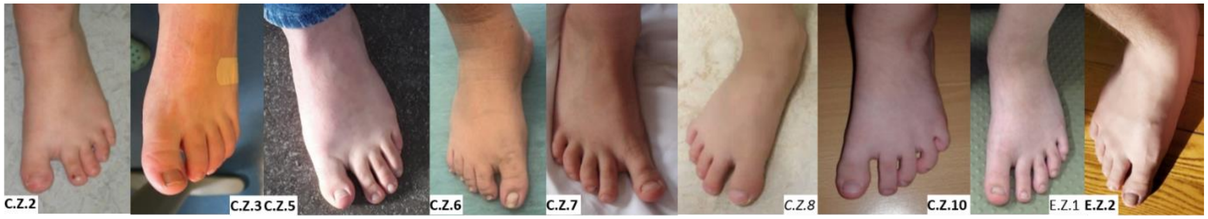
C.Z.2
C.Z.3
C.Z.5
C.Z.6
C.Z.7
C.Z.8
C.Z.10
E.Z.1
E.Z.2

B

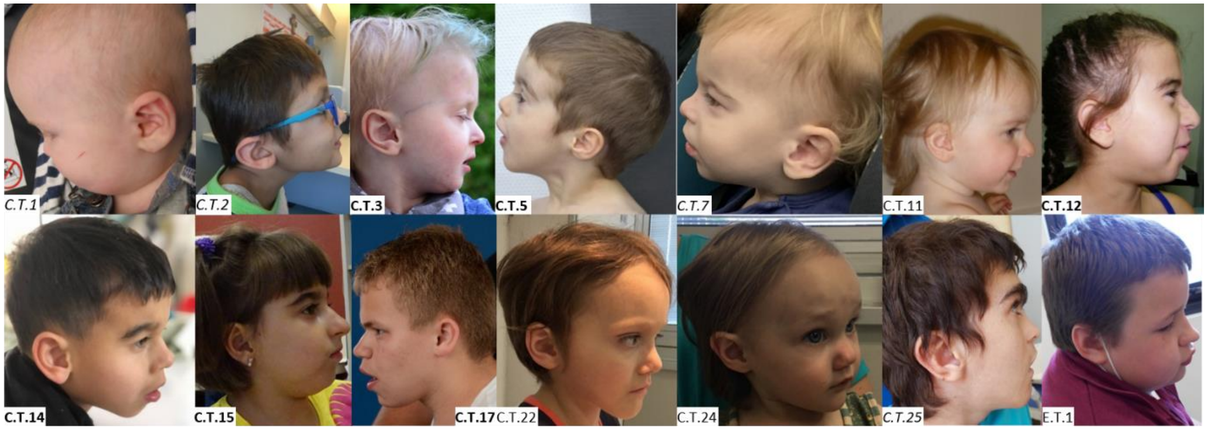
C.T.1
C.T.2
C.T.3
C.T.5
C.T.7
C.T.11
C.T.12
C.T.14
C.T.15
C.T.17
C.T.22
C.T.24
C.T.25
E.T.1

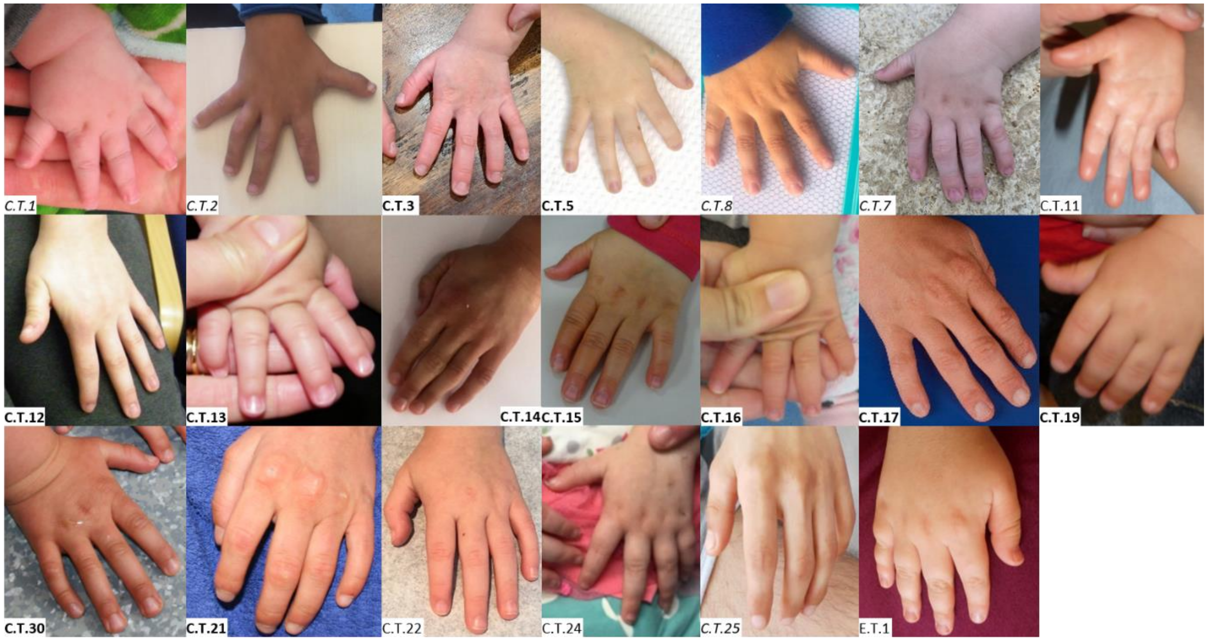
C.T.1
C.T.2
C.T.3
C.T.5
C.T.8
C.T.7
C.T.11
C.T.12
C.T.13
C.T.14
C.T.15
C.T.16
C.T.17
C.T.19
C.T.30
C.T.21
C.T.22
C.T.24
C.T.25
E.T.1

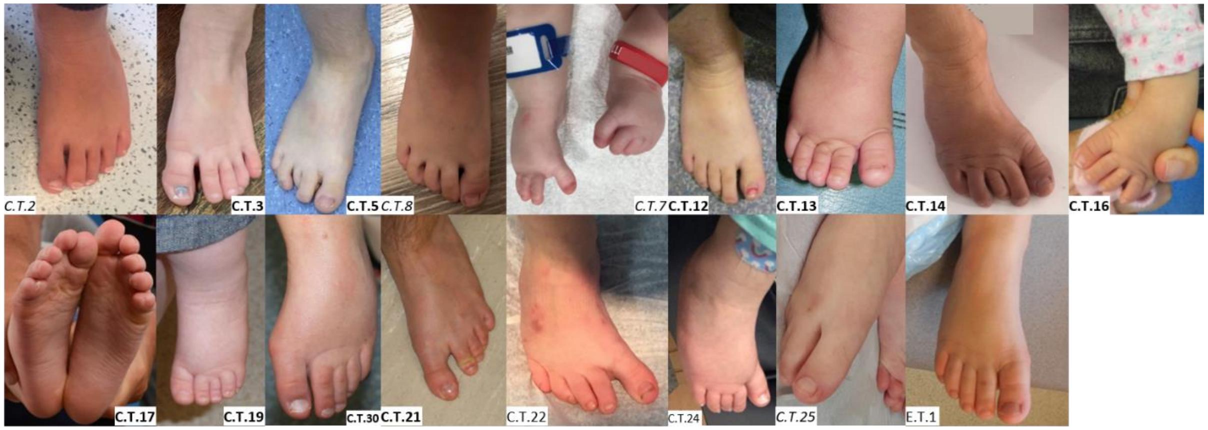
C.T.2
C.T.3
C.T.5
C.T.8
C.T.7
C.T.12
C.T.13
C.T.14
C.T.16
C.T.17
C.T.19
C.T.30
C.T.21
C.T.22
C.T.24
C.T.25
E.T.1

C

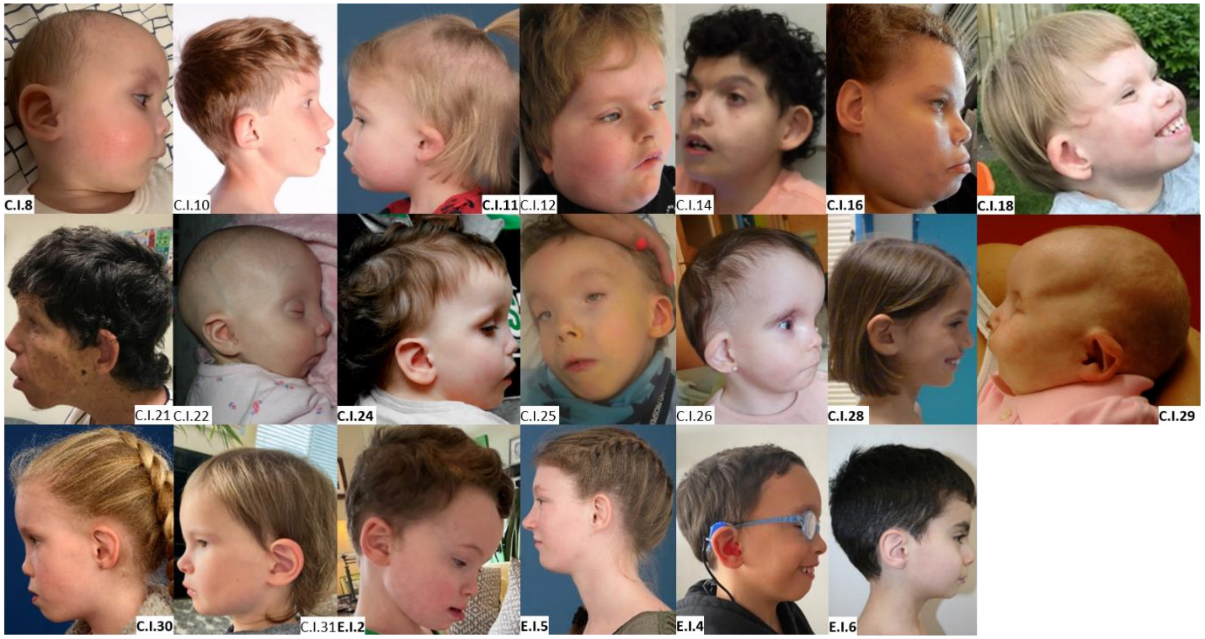
C.I.8
C.I.10
C.I.11
C.I.12
C.I.14
C.I.16
C.I.18
C.I.21
C.I.22
C.I.24
C.I.25
C.I.26
C.I.28
C.I.29
C.I.30
C.I.31
E.I.2
E.I.5
E.I.4
E.I.6

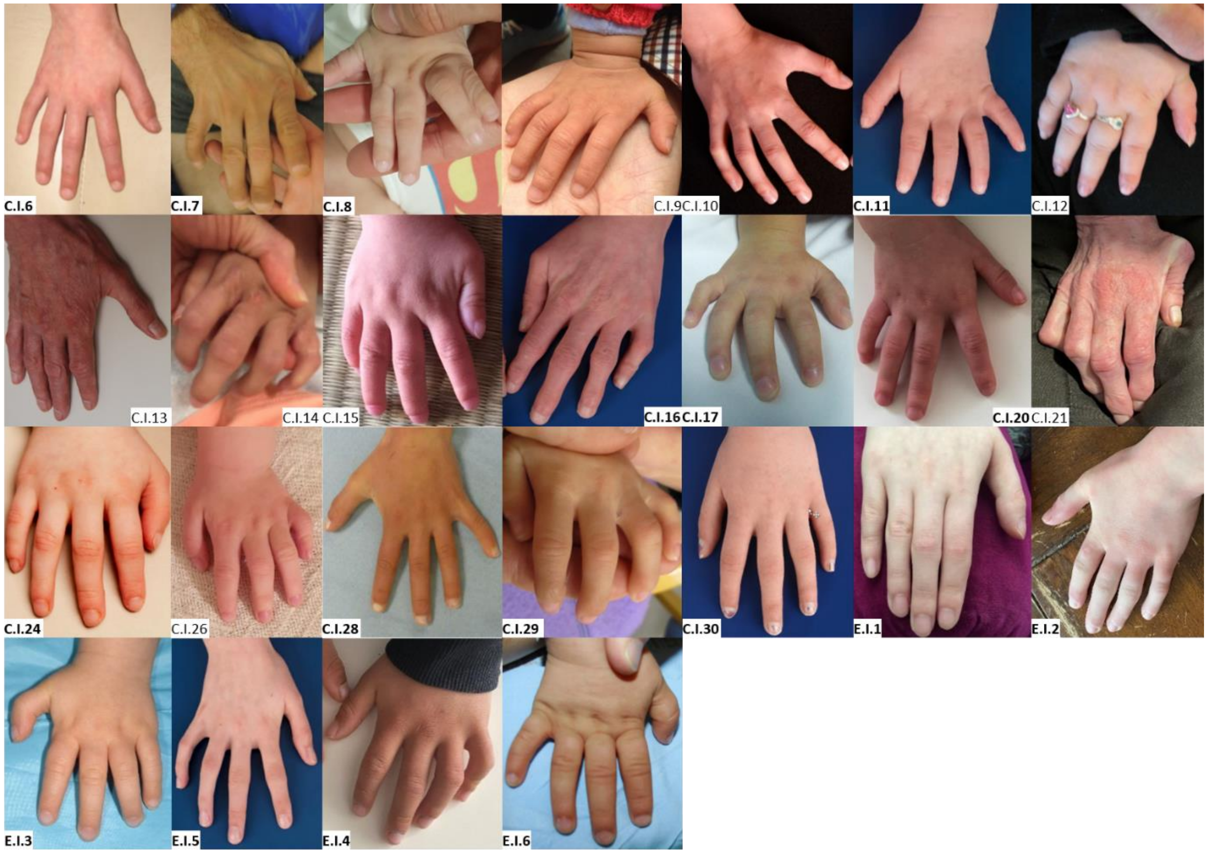
C.I.6
C.I.7
C.I.8
C.I.9
C.I.10
C.I.11
C.I.12
C.I.13
C.I.14
C.I.15
C.I.16
C.I.17
C.I.20
C.I.21
C.I.24
C.I.26
C.I.28
C.I.29
C.I.30
E.I.1
E.I.2
E.I.3
E.I.5
E.I.4
E.I.6

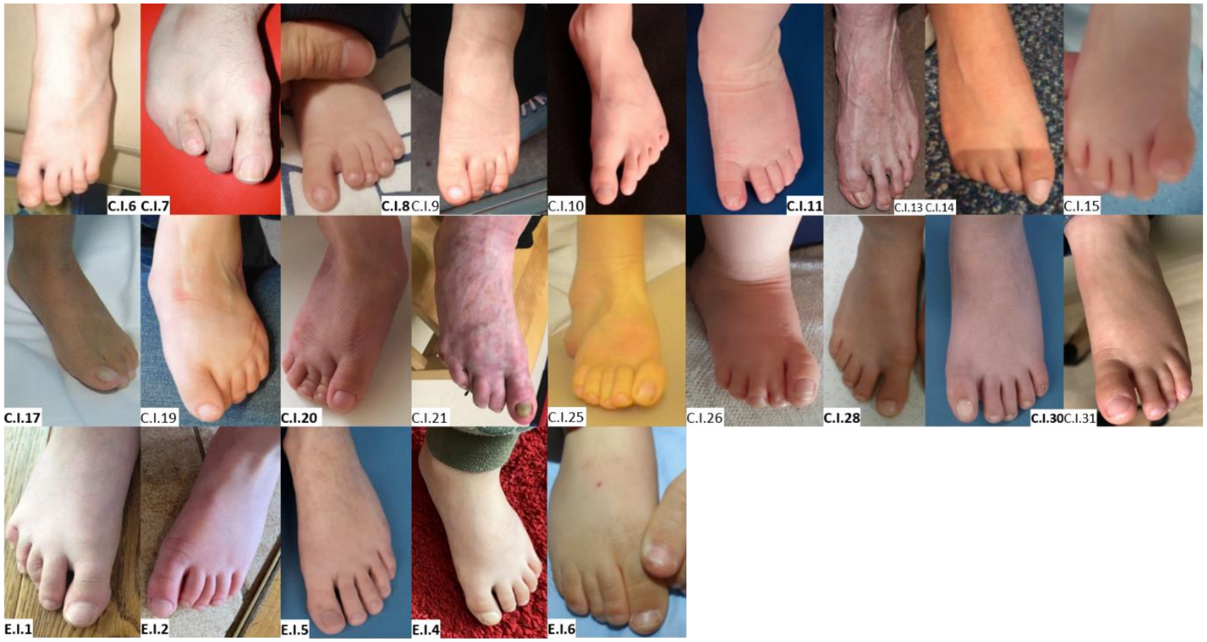
C.I.6
C.I.7
C.I.8
C.I.9
C.I.10
C.I.11
C.I.13
C.I.14
C.I.15
C.I.17
C.I.19
C.I.20
C.I.21
C.I.25
C.I.26
C.I.28
C.I.30
C.I.31
E.I.1
E.I.2
E.I.5
E.I.4
E.I.6

D

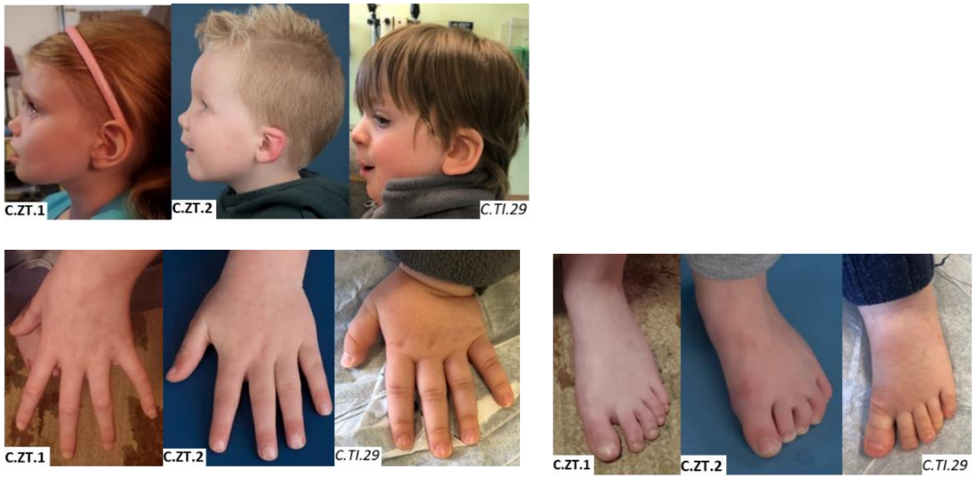
C.ZT.1
C.ZT.2
C.TI.29
C.ZT.1
C.ZT.2
C.TI.29
C.ZT.1
C.ZT.2
C.TI.29

Table S1. Summary of the 3-step process for the selection of probes

Episignature	Number of probes after the first step	Number of probes after the second step	Cut-off value for correlation
MKHK_ZZ	1000	333	0.85
MKHK_TAZ2	900	225	0.75
MKHK_ID4	900	225	0.75

Table S2. Morphological characteristics of individuals with a confirmed MKHK methylation profile or episignature (in ID4) per MKHK subtype.

ZZ, zinc finger ZZ-type; TAZ2, zinc finger TAZ-type; ID4, first α-helix of the fourth intrinsically disordered region of CBP/p300.

	**MKHK-ZZ (n=9)**	**MKHK-TAZ2 (n=14)**	**MKHK-ID4 (n=21)**
**Sparse hair**	0/9	3/12 (25%)	10/19 (53%)
**Prominent forehead**	2/9 (22%)	4/13 (31%)	15/20(75%)
**Face, square (FS) or flat (FF)**	FF 1/9 (11%)<br>FS 1/9 (11%)	FF 1/13 (8%)<br>FS 2/13 (15%)	FF 6/20 (30%)<br>FS 6/20 (30%)
**Thick eyebrows**	5/9 (56%)	7/13 (54%)	6/21 (29%)
**Flared eyebrows**	5/9 (56%)	0/13	0/18
**Telecanthi (T)/ epicanthi (E)**	T 2/9 (22%)<br>E 2/9 (22%)	T 2/14 (14%)<br>E 5/14 (36%)	T 7/21 (33%)<br>E 7/21 (33%)
**Palpebral fissures Up (U)- or downslanted (D)**	U 0/7<br>D 1/7 (14%)	U 1/13 (8%)<br>D 2/13 (15%)	U 15/21 (71%)<br>D 0/21
**Short palpebral fissures**	1/9 (11%)	2/14 (14%)	15/21 (71%)
**Ptosis/ Blepharophimosis**	4/8 (50%)	5/11 (45%)	15/21 (71%)
**Long eyelashes**	3/9 (33%)	5/12 (42%)	5/20 (25%)
**Ears Low-set (L) / Short (S)**	L 0/8<br>S 0/8	L 2/9 (22%)<br>S 1/9 (11%)	L 6/19 (32%)<br>S 1/19 (5%)
**Protruding ears (upper part)**	0/9	3/12 (25%)	13/20 (65%)
**Overfolded helix**	1/8 (13%)	1/11 (9%)	8/19 (42%)
**Absent earlobe**	0/8	1/11 (9%)	2/19 (10%)
**Full cheeks**	4/9 (44%)	3/14 (21%)	10/21 (48%)
**Malar flattening**	0/9	1/12 (8%)	8/18 (44%)
**Short nose**	2/9 (22%)	2/14 (14%)	11/21 (52%)
**Depressed nasal bridge**	1/9 (11%)	0/12	9/18 (50%)
**Narrow nasal bridge**	1/9 (11%)	2/14 (14%)	2/21 (9%)
**Depressed nasal ridge**	0/9	1/12 (7%)	10/18 (56%)
**Broad nasal tip**	3/9 (33%)	8/14 (57%)	12/21 (57%)
**Short columella**	0/8	3/13 (23%)	14/21 (67%)
**Anteverted nares**	1/9 (11%)	4/14 (29%)	11/21 (52%)
**Underdeveloped alae nasi**	0/9	1/13 (8%)	1/21 (5%)
**Philtrum Short (S)/Long (L)/Deep (D)**	S 1/9 (11%)<br>L 1/9 (11%)<br>D 0	S 0/13<br>L 5/13 (38%)<br>D 1/13 (8%)	S 1/21 (5%)<br>L 13/21 (62%)<br>D 4/21 (19%)
**Everted vermilion of upper lip**	1/9 (11%)	2/13 (15%)	4/21 (19%)
**Thin vermilion of upper lip**	5/9 (56%)	4/13 (31%)	6/21 (29%)
**High palate**	6/7 (86%)	1/10 (10%)	10/17 (59%)
**Missing teeth**	2/8 (25%)	0/8	3/10 (30%)
**Retro- or micrognathia**	1/9 (11%)	5/12 (42%)	5/19 (26%)
**Ulnar deviation of finger(s)**	0/7	0/13	3/20 (15%)
**Clinodactyly fifth finger**	1/9 (11%)	2/11 (18%)	8/20 (40%)
**Tapering fingers**	0/6	0/13	2/20 (10%)
**Prominent digital pads**	0/6	1/8 (13%)	4/15 (27%)
**Sandal gap**	3/6 (50%)	3/11 (27%)	5/18 (28%)
**Overlapping toes**	0/6	3/13 (23%)	6/19 (32%)
**Halluces Broad (B)/Narrow (N)**	0/5	B 2/12 (17%)<br>N 1/12 (8%)	B 3/18 (17%)<br>N 1/18 (6%)
**Fibular deviation distal phalanx halluces**	0/6	2/12 (17%)	8/20 (40%)
**Cutaneous partial syndactyly of toes**	0/6	2/12 (17%)	5/18 (28%)

Table S3 (see excel file). Clinical characteristics and morphological features of the presently described individuals with a variant in the MKHK region of *CREBBP* and *EP300*.

The columns of individuals for whom a DNA sample was available are green colored if the diagnosis of the MKHK-subtype was confirmed by episignature testing, and orange colored in case the episignature result did not show any MKHK episignature. [a] Menke et al. 2016[1]; [b] Menke et al. 2018[2]; [c] Angius et al. 2019[3]; [d] Banka et al. 2019.[4] ADHD, attention deficit hyperactivity disorder; ASD, autism spectrum disorder; ASDsf: some features of ASD; ASd: atrial septal defect; BMI, body mass index; DMII, diabetes mellitus type 2; Dpt, diopters; eTSH, elevated thyroid stimulating hormone; GE, gastro-esophageal; GHD, growth hormone deficiency; GI, gastrointestinal; G-tube, gastric tube; ID, intellectual disability; IVC, inferior vena cava; LT, low testosterone level; n.a.; not available; OD, right eye; OFC, occipito-frontal circumference; OS, left eye; PDA, patent ductus arteriosis; PEG, percutaneous gastrostomy; PE-tubes, pressure equalizing tubes; PFO, patent foramen ovale; PVL, periventricular leucomalacia; PVS, Pulmonary valve stenosis; RRTI, recurrent respiratory tract infections; RUTI, recurrent urinary tract infections; SDS, standard deviation score; TSH, thyroid stimulating hormone; TVP, tricuspid valve prolapse; VSD, ventricle septal defect; VUR, vesicoureteral reflux.

Table S4 (see excel file). Genotypes, episignatures and in silico prediction scores of all presently described individuals with a variant in the MKHK region of CREBBP (NM_004380.2) or EP300 (NM_001429.3).

ZZ, Zinc-finger ZZ-type; TAZ2, Zinc-finger TAZ-type; ID4, first α-helix of the fourth intrinsically disordered region. [a]GnomAD: number represents how many times the allele was seen in gnomAD (Genome Aggregation Database) with '.' representing not seen, which is slightly different than '0' (seen but filtered out in the final gnomAD population). CADD, Combined Annotation Dependent Depletion; REVEL, Rare Exome Variant Ensemble Learner; SIFT, Sorting Intolerant From Tolerant; PolyPhen, Polymorphism Phenotyping; MPC, 'Missense badness, PolyPhen-2 and Constraint'.

Table S5. List of identified differentially methylated regions.

Chromosome	Start	End	Width	Number of CpGs	Stouffer	Fisher	Maximum difference	Mean difference	Overlapping gene(s)
**chr17**	40822424	40824241	1818	8	6,98E-77	2,55E-73	0,22913	0,167478	PLEKHH3
**chr6**	26195488	26197606	2119	8	9,12E-36	1,53E-57	-0,25391	-0,10266	AL031777.2,H3C4
**chr6**	27858545	27859478	934	6	1,67E-12	1,07E-27	-0,1179	-0,05217	H3C12
**chr7**	27169136	27171391	2256	21	1,15E-21	3,8E-26	-0,13849	-0,0732	HOXA3,HOXA-AS2,HOXA4,AC004080.6, HOXA-AS3
**chr5**	42756102	42757815	1714	11	1,26E-13	4,4E-24	-0,1223	-0,05729	CCDC152
**chr6**	27782126	27783336	1211	6	1,53E-11	1,74E-19	-0,1646	-0,06806	H2AC14,H2BC14
**chr17**	14205882	14207968	2087	15	1,14E-13	4,14E-19	-0,12428	-0,05383	HS3ST3B1,AC005224.3
**chr5**	1,5E+08	1,5E+08	1504	10	2,12E-11	1,05E-14	0,116828	0,05455	CAMK2A
**chr19**	11517079	11517953	875	7	5,16E-07	1,64E-14	0,142259	0,066341	RGL3
**chr12**	58131345	58133008	1664	9	5,13E-09	5,41E-14	-0,18415	-0,08062	AGAP2,TSPAN31
**chr1**	1,57E+08	1,57E+08	1268	6	5,09E-12	8,5E-14	-0,14	-0,07838	TTC24
**chr6**	26224668	26226256	1589	11	8,64E-12	1,97E-13	-0,1307	-0,0787	H3C6
**chr12**	1641924	1643253	1330	5	2,4E-06	7,74E-12	0,108645	0,056643	WNT5B
**chr9**	98314632	98315816	1185	6	2,28E-06	4,09E-11	-0,109	-0,05646	
**chr1**	27675587	27676652	1066	7	3,33E-12	4,22E-11	-0,09359	-0,06941	SYTL1
**chr10**	70321243	70322874	1632	9	4,52E-06	1,54E-10	-0,11566	-0,05487	TET1
**chr19**	55660514	55661872	1359	6	7,63E-10	2,9E-10	0,156144	0,086835	TNNT1
**chr1**	2,36E+08	2,36E+08	785	6	5,47E-09	2,16E-09	0,109219	0,064921	GNG4
**chr22**	42394048	42395340	1293	11	1,72E-05	4,77E-09	0,115519	0,060151	SEPTIN3,WBP2NL
**chr3**	1,94E+08	1,94E+08	691	7	2,62E-10	9,62E-09	-0,11353	-0,08543	LINC00887
**chr1**	11708564	11709491	928	5	5,48E-07	1,85E-08	0,093936	0,051314	FBXO2
**chr3**	42846594	42847116	523	5	1,09E-06	2,84E-08	-0,11095	-0,05701	AC099329.1,ACKR2
**chr3**	1,41E+08	1,41E+08	544	6	1,94E-08	2,86E-08	-0,0906	-0,06869	ZBTB38
**chr5**	79552470	79553606	1137	5	1,92E-08	4,09E-08	-0,11464	-0,069	
**chr5**	83016630	83017921	1292	11	9,31E-07	4,18E-08	-0,09717	-0,05656	HAPLN1
**chr17**	46719276	46720386	1111	5	6,27E-08	5,18E-08	0,083397	0,055208	AC103702.2
**chr1**	2,35E+08	2,35E+08	645	5	1,48E-06	1,18E-07	-0,08859	-0,05036	
**chr1**	6514605	6516324	1720	5	9,55E-07	2,09E-07	0,096204	0,061211	ESPN
**chr2**	2,43E+08	2,43E+08	481	5	4,77E-06	8,9E-07	0,081801	0,05709	
**chr12**	54412506	54413936	1431	8	6,44E-07	2,07E-06	0,099361	0,065565	AC012531.3,HOXC6,HOXC4,AC012531.2, AC012531.1
**chr10**	21798341	21799395	1055	6	2,59E-06	7,25E-06	-0,09209	-0,06279	
**chr17**	17109640	17110641	1002	11	6,46E-06	7,78E-06	0,097594	0,066414	MPRIP,PLD6,AC055811.2
**chr18**	22907319	22908222	904	5	5,9E-05	8,46E-06	-0,12209	-0,07057	ZNF521
**chr1**	63249197	63249705	509	7	0,002966	9,24E-06	0,113212	0,053852	
**chr1**	86043795	86044230	436	6	2,59E-05	1,07E-05	-0,09664	-0,06059	DDAH1,AC092807.4,AC092807.3
**chr9**	35791475	35792382	908	6	9,94E-05	1,16E-05	-0,10736	-0,05536	NPR2
**chr15**	99408636	99409957	1322	10	2,89E-06	1,37E-05	0,068171	0,053442	IGF1R
**chr19**	36004632	36005395	764	6	4,15E-05	1,84E-05	0,091674	0,060381	
**chr2**	1,64E+08	1,64E+08	716	7	1,17E-06	1,95E-05	-0,09102	-0,06841	
**chr5**	1245669	1246795	1127	6	2,37E-05	2,18E-05	0,094337	0,063162	SLC6A18

**chr17**	78734663	78735596	934	6	3,78E-05	3,15E-05	0,086993	0,054553	RPTOR
**chr1**	27683139	27683912	774	7	6,69E-06	3,29E-05	0,064924	0,052358	MAP3K6
**chr19**	51165207	51165845	639	5	3,59E-05	3,3E-05	0,083174	0,061784	SHANK1
**chr11**	1769152	1770066	915	9	7,68E-05	3,45E-05	0,079182	0,051487	IFITM10,AC068580.4
**chr19**	50249464	50249927	464	5	0,000347	4,02E-05	0,12787	0,07806	TSKS
**chr4**	4763259	4763753	495	7	2,36E-05	6E-05	-0,11503	-0,05714	STX18-AS1
**chr1**	1289806	1291064	1259	8	5,16E-06	6,02E-05	0,097064	0,058265	MXRA8
**chr11**	18477153	18478135	983	7	8,57E-05	6,3E-05	0,12583	0,062944	LDHAL6A
**chr11**	45671208	45672248	1041	5	8,1E-06	6,46E-05	0,087335	0,063194	CHST1
**chr12**	1,31E+08	1,31E+08	759	7	0,000119	8,38E-05	0,109142	0,054137	STX2
**chr9**	1,34E+08	1,34E+08	626	5	4,07E-05	9,53E-05	-0,08873	-0,07043	
**chr19**	49223814	49224454	641	5	3,55E-05	9,9E-05	-0,0969	-0,0565	RASIP1
**chr1**	6187633	6188136	504	5	2,65E-05	0,000131	0,099593	0,068783	CHD5
**chr7**	27127448	27128169	722	6	1,99E-05	0,00015	0,067895	0,050098	
**chr1**	2,44E+08	2,44E+08	1119	5	2,29E-05	0,000164	0,070203	0,052386	ZBTB18
**chr17**	9549496	9550545	1050	8	0,000162	0,000171	-0,09224	-0,05537	USP43,AC118755.1
**chr3**	1,84E+08	1,84E+08	634	7	0,000308	0,000185	0,124694	0,051593	CHRD
**chr10**	75533025	75533431	407	5	0,000418	0,000299	0,102109	0,057383	FUT11
**chr1**	27718221	27718858	638	5	0,000827	0,0003	0,08923	0,050069	
**chr5**	2537210	2537834	625	8	0,000111	0,000382	0,087529	0,053924	
**chr3**	1,6E+08	1,6E+08	480	6	3,71E-05	0,000404	-0,08339	-0,0682	IQCJ-SCHIP1,SCHIP1
**chr4**	1,75E+08	1,75E+08	208	5	0,00032	0,000408	-0,09371	-0,06334	LINC02269,AC106895.2
**chr1**	19971709	19972777	1069	8	5,83E-05	0,00061	-0,10402	-0,06221	NBL1,MICOS10-NBL1
**chr8**	38831681	38832728	1048	6	0,001872	0,001269	-0,07691	-0,05204	HTRA4
**chr11**	834676	835377	702	5	0,004198	0,001496	0,113156	0,061579	CD151
**chr19**	50861774	50862121	348	5	0,000291	0,001703	0,080581	0,060911	NR1H2,NAPSA
**chr6**	1,64E+08	1,64E+08	715	6	0,000407	0,001772	-0,09124	-0,06431	AL078602.1
**chr12**	9217079	9217907	829	11	0,001172	0,002018	-0,10539	-0,06154	LINC00612,A2M-AS1
**chr11**	396686	397486	801	5	0,000753	0,002831	0,095488	0,070649	PKP3
**chr1**	1,12E+08	1,12E+08	474	6	0,001355	0,005106	0,063995	0,052387	C1orf162
**chr1**	47900197	47900327	131	5	0,003204	0,006846	0,079071	0,062555	FOXD2-AS1
**chr22**	38244746	38244902	157	5	0,001669	0,007427	0,070153	0,055552	ANKRD54,EIF3L
**chr2**	20441905	20442210	306	5	0,005672	0,007602	0,078123	0,050274	
**chr10**	1,01E+08	1,01E+08	519	5	0,001764	0,008912	-0,09154	-0,06371	

Start, genomic position of the region's start point; End, genomic position of the region's end point; Width, the width of the region; Stouffer, Stouffer summary transform of the individual CpG false discovery rates (FDRs); Fisher, Fisher combined probability transform of the individual CpG FDRs; Maximum difference: Maximum methylation difference within the DMR; Mean difference: Mean methylation difference across the DMR.

References

1. Menke, L.A., van Belzen, M.J., Alders, M., Cristofoli, F., Ehmke, N., Fergelot, P., Foster, A., Gerkes, E.H., Hoffer, M.J., Horn, D., et al. (2016). CREBBP mutations in individuals without Rubinstein-Taybi syndrome phenotype. Am J Med Genet A *170*, 2681-2693. 10.1002/ajmg.a.37800.
2. Menke, L.A., Gardeitchik, T., Hammond, P., Heimdal, K.R., Houge, G., Hufnagel, S.B., Ji, J., Johansson, S., Kant, S.G., Kinning, E., et al. (2018). Further delineation of an entity caused by CREBBP and EP300 mutations but not resembling Rubinstein-Taybi syndrome. Am J Med Genet A *176*, 862-876. 10.1002/ajmg.a.38626.
3. Angius, A., Uva, P., Oppo, M., Persico, I., Onano, S., Olla, S., Pes, V., Perria, C., Cuccuru, G., Atzeni, R., et al. (2019). Confirmation of a new phenotype in an individual with a variant in the last part of exon 30 of CREBBP. Am J Med Genet A *179*, 634-638. 10.1002/ajmg.a.61052.
4. Banka, S., Sayer, R., Breen, C., Barton, S., Pavaine, J., Sheppard, S.E., Bedoukian, E., Skraban, C., Cuddapah, V.A., and Clayton-Smith, J. (2019). Genotype-phenotype specificity in Menke-Hennekam syndrome caused by missense variants in exon 30 or 31 of CREBBP. Am J Med Genet A *179*, 1058-1062. 10.1002/ajmg.a.61131.
