# Supplementary material for: Menke-Hennekam syndrome; delineation of domain-specific subtypes with distinct clinical and DNA methylation profiles
Source: HGG Adv. 2024 Mar 29;5(3):100287. doi: 10.1016/j.xhgg.2024.100287 (PMC11040166; doi:10.1016/j.xhgg.2024.100287)
Supplement: Document S2. Article plus supplemental information [file mmc4.pdf]

HGG Advances

ARTICLE

Menke-Hennekam syndrome; delineation of domain-specific subtypes with distinct clinical and DNA methylation profiles

Sadegheh Haghshenas,[1,22] Hidde J. Bout,[2,22] Josephine M. Schijns,[2] Michael A. Levy,[1] Jennifer Kerkhof,[1] Pratibha Bhai,[1] Haley McConkey,[1] Zandra A. Jenkins,[3] Ella M. Williams,[3] Benjamin J. Halliday,[3] Sylvia A. Huisman,[2,4] Peter Lauffer,[5] Vivian de Waard,[6] Laura Witteveen,[2] Siddharth Banka,[7,8] Angela F. Brady,[9] Elena Galazzi,[10] Julien van Gils,[11] Anna C.E. Hurst,[12] Frank J. Kaiser,[13,14] Didier Lacombe,[11] Antonio F. Martinez-Monseny,[15] Patricia Fergelot,[11] Fabíola P. Monteiro,[16] Ilaria Parenti,[13] Luca Persani,[10,17] Fernando Santos-Simarro,[18,19] Brittany N. Simpson,[20] MKHK Research Consortium, Mariëlle Alders,[5] Stephen P. Robertson,[3] Bekim Sadikovic,[1,21,*] and Leonie A. Menke[2,23,*]

Summary

CREB-binding protein (CBP, encoded by *CREBBP*) and its paralog E1A-associated protein (p300, encoded by *EP300*) are involved in histone acetylation and transcriptional regulation. Variants that produce a null allele or disrupt the catalytic domain of either protein cause Rubinstein-Taybi syndrome (RSTS), while pathogenic missense and in-frame indel variants in parts of exons 30 and 31 cause phenotypes recently described as Menke-Hennekam syndrome (MKHK). To distinguish MKHK subtypes and define their characteristics, molecular and extended clinical data on 82 individuals (54 unpublished) with variants affecting CBP ($n = 71$) or p300 ($n = 11$) (NP_004371.2 residues 1,705–1,875 and NP_001420.2 residues 1,668–1,833, respectively) were summarized. Additionally, genome-wide DNA methylation profiles were assessed in DNA extracted from whole peripheral blood from 54 individuals. Most variants clustered closely around the zinc-binding residues of two zinc-finger domains (ZZ and TAZ2) and within the first α helix of the fourth intrinsically disordered linker (ID4) of CBP/p300. Domain-specific methylation profiles were discerned for the ZZ domain in CBP/p300 (found in nine out of 10 tested individuals) and TAZ2 domain in CBP (in 14 out of 20), while a domain-specific diagnostic episignature was refined for the ID4 domain in CBP/p300 (in 21 out of 21). Phenotypes including intellectual disability of varying degree and distinct physical features were defined for each of the regions. These findings demonstrate existence of at least three MKHK subtypes, which are domain specific (MKHK-ZZ, MKHK-TAZ2, and MKHK-ID4) rather than gene specific (*CREBBP*/*EP300*). DNA methylation episignatures enable stratification of molecular pathophysiologic entities within a gene or across a family of paralogous genes.

Introduction

CREB-binding protein (CBP, encoded by *CREBBP*; OMIM: 600140) and its paralog E1A-associated protein (p300, encoded by *EP300*; OMIM: 602700) are histone acetyltransferases and important cofactors for transcription.[1,2] Variants that produce a null allele or impair their catalytic function cause Rubinstein-Taybi syndrome (RSTS; OMIM: 180849 and: 613684),[1–3] a well-known entity characterized by a characteristic face, broad thumbs, broad big toes, short stature, and intellectual disability.[4] In 2016, we reported 11 individuals with a variant in parts of exons 30 and 31 located within the C-terminal region of *CREBBP* who did not resemble the classic RSTS phenotype.[5] In 2018, we reported an additional 11 individuals with variants in this region of *CREBBP*, as well as two individuals

[1]Verspeeten Clinical Genome Centre, London Health Sciences Centre, London ON N6A 5W9, Canada; [2]Department of Pediatrics, Emma Children's Hospital, Amsterdam UMC, University of Amsterdam, Amsterdam Reproduction and Development Research Institute, 1105 Amsterdam, AZ, the Netherlands; [3]Department of Women's and Children's Health, Dunedin School of Medicine, University of Otago, Dunedin 9016, New Zealand; [4]Zodiak, Prinsenstichting, Purmerend, JE 1444, the Netherlands; [5]Department of Human Genetics, Amsterdam UMC, University of Amsterdam, Amsterdam Reproduction and Development Research Institute, Amsterdam 1105 AZ, the Netherlands; [6]Department of Medical Biochemistry, Amsterdam UMC, University of Amsterdam, Amsterdam Cardiovascular Sciences, Amsterdam, AZ 1105, the Netherlands; [7]Division of Evolution, Infection and Genomics, School of Biological Sciences, Faculty of Biology, Medicine and Health, University of Manchester, Manchester M13 9WL, UK; [8]Manchester Centre for Genomic Medicine, Saint Mary's Hospital, Manchester University NHS Foundation Trust, Manchester M13 9WL, UK; [9]North West Thames Regional Genetics Service, Northwick Park Hospital, Harrow HA1 3UJ, UK; [10]Department of Endocrine & Metabolic Diseases, San Luca Hospital, IRCCS Istituto Auxologico Italiano, 20100 Milan, Italy; [11]Centre Hospitalier Universitaire Bordeaux, 33404 Bordeaux, France; [12]Department of Genetics, University of Alabama, Birmingham, AL 35294-0024, USA; [13]Institute of Human Genetics, University of Duisburg-Essen, 45122 Essen, Germany; [14]Center for Rare Diseases, University Hospital Essen, 45122 Essen, Germany; [15]Genètica Clínica, Servei de Medicina Genètica i Molecular, Hospital Sant Joan de Déu, 08950 Barcelona, Spain; [16]Alameda dos Maracatins, Indianópolis, São Paulo 1435, Brazil; [17]Department of Medical Biotechnology and Translational Medicine, University of Milan, 20100 Milan, Italy; [18]Institute of Medical and Molecular Genetics (INGEMM), Hospital Universitario La Paz, IdiPAZ, CIBERER, ISCIII, 28029 Madrid, Spain; [19]Unit of Molecular Diagnostics and Clinical Genetics, Hospital Universitari Son Espases, Health Research Institute of the Balearic Islands (IdISBa), 07120 Palma, Spain; [20]Department of Pediatrics, Division of Human Genetics, Cincinnati Children's Hospital Medical Center, University of Cincinnati School of Medicine, Cincinnati, OH 45206, USA; [21]Department of Pathology and Laboratory Medicine, Western University, London, ON N6A3K7, Canada
[22]These authors contributed equally
[23]Lead contact
*Correspondence: bekim.sadikovic@lhsc.on.ca (B.S.), l.a.menke@amsterdamumc.nl (L.A.M.)
https://doi.org/10.1016/j.xhgg.2024.100287.
© 2024 The Authors. This is an open access article under the CC BY license (http://creativecommons.org/licenses/by/4.0/).

with variants in the homologous region of *EP300*, who did not show the characteristics typical for RSTS.[6]

Subsequently, individuals carrying these variants were established as having an entity distinct from RSTS, recently described as Menke-Hennekam syndrome (MKHK1 and MKHK2; OMIM: 618332 and 618333, for variants in *CREBBP* and *EP300*, respectively).[7]

The MKHK region of CBP/p300 spans two zinc-finger domains (ZZ[8] and TAZ2[9]) and the first α helix of the fourth intrinsically disordered linker (ID4).[6,10,11] Although OMIM categorized MKHK into type 1 (OMIM: 618332) and 2 (OMIM: 618333) for variants in *CREBBP* and *EP300*, respectively, we[6,7,12] and others[13,14] hypothesized that MKHK may in fact consist of multiple domain-specific rather than gene-specific subtypes. We previously showed that individuals with variants in ID4 of CBP/p300 seemed to have a specific phenotype,[6] which was subsequently substantiated by us with the discovery of a genome-wide DNA methylation pattern ("episignature") in these individuals.[11] Genome-wide DNA methylation episignatures provide a sensitive and specific biomarker for an increasing number of Mendelian disorders.[11]

In this study, we report molecular, clinical, and morphological data from a large number of individuals with MKHK, and we describe two novel MKHK methylation profiles and a refined MKHK-ID4 episignature, enabling us to more accurately define and delineate each of the MKHK subtypes.

Subjects, material, and methods

Phenotype and genotype

Individuals were eligible for inclusion if they had a CBP/p300 (NP_004371.2 and NP_001420.2, respectively) variant within the area known to cause MKHK, consisting of the ZZ and TAZ2 domains, the first α helix of ID4, and the two areas between them. The boundaries of the ZZ (CBP, residues 1,705–1,745; p300, residues 1,668–1,708) and TAZ2 (CBP, 1,772–1,840; p300, 1,735–1,803) domains were defined by the 2022/06 NCBI consensus (CDD: 239077 and CDD: 426615), and the region of the first α helix of ID4 was defined according to Piai et al.[10] (CBP, residues 1,852–1,875, the homologous residues in p300 being 1,810–1,833). All individuals received a study number based on the affected gene (C or E representing *CREBBP* and *EP300*) and domain (Z, T, I, ZT, and TI representing ZZ, TAZ2, ID4, the region between ZZ and TAZ2, and the region between TAZ2 and ID4, respectively) and a unique number. The cohort consisted of (1) previously reported individuals,[5–7,12] (2) individuals who had been referred to us after the previous publications, and (3) individuals who were ascertained through ClinVar[15] or Decipher.[16] Clinical and genetic data were gathered using a standardized patient report form, which was completed by the local clinical geneticist/physician. Facial and distal limb morphology were scored by one expert (L.A.M.). Study procedures were approved by the medical ethics committee of Amsterdam UMC (NL65113.018.18; 2018_109#B2018478a) and the Western University Research Ethics Board (REB 106302). Written informed consent for publication of data, clinical pictures, and/or the use of DNA for the DNA methylation analysis was obtained from parents/legal guardians of all individuals.

Population allele frequency data from the gnomAD project (v2.1.1),[17] evolutionary conservation score PhyloP (100-way vertebrate alignment),[18] and pre-computed *in silico* variant effect predictor scores were annotated with BCFtools annotate (v1.12).[19] *In silico* variant effect predictors were chosen based on frequent citation in previous literature (SIFT[20] and PolyPhen2-HVAR[21] and CADD[22])[23] or performance in ensemble predictor comparison studies (REVEL[24] and MPC[25]).[26,27]

Three-dimensional protein structures of CBP and p300 (including hydrogen bonds and zinc ions) were predicted using AlphaFold and AlphaFill (https://alphafill.eu/v1/aff/Q92793 and https://alphafill.eu/v1/aff/Q09472). In PyMOL, the HAT, ZZ, and TAZ2 domains were colored according to the NCBI consensuses, and the first α helix of ID4 according to Piai et al.,[10] based on the previously mentioned regional boundaries. All variant residues in this cohort (except for deletions and duplications) were then highlighted on the structure, along with predicted hydrogen bonds between the ID4 helix and HAT domain.

The interpretation of sequence variants was done according to The American College of Medical Genetics and Genomics (ACMG) criteria.[28]

DNA methylation analysis

DNA derived from peripheral blood samples of individuals were processed using Illumina Infinium EPIC bead chip arrays (San Diego, CA), covering over 860,000 human genome CpG sites as described previously.[29] In summary, using R version 4.0.5 and minfi package version 1.40.0, methylated and unmethylated signal intensities were normalized with background correction.[30] Arrays with failed probe rates above 5% were excluded from the analysis. Probes were removed if known to contain single-nucleotide polymorphisms (SNPs) at or near CpG interrogation or single-nucleotide extension sites, located on X and Y chromosomes, known to cross-react with chromosomal locations other than their target regions, or having a detection p value >0.01. Rounds of principal-component analysis (PCA) were performed and outliers (samples with either their first or second PCA components not within three standard deviations of the corresponding component) were removed on each round, until no further outliers were detected in the first two components of the PCA.

Selection of matched controls

Control samples matched by age, sex, and array type were selected from the EpiSign Knowledge Database (EKD).[11] Based on sample sizes of the groups of individuals with variants in the ZZ and TAZ2 domains, and ID4 region, the

number of control samples selected were 54, 60, and 63, respectively.

Methylation profiling for the three MKHK protein regions (ZZ, TAZ2, ID4)

For each probe, the methylation level (β value), was calculated as the methylated signal intensity divided by the sum of methylated and unmethylated signal intensities. These values were then converted into M values using logit transformation to obtain homoscedasticity for linear modeling. To assess episignatures for the three protein regions/domains, the following process was performed separately for each pair of case samples and matched controls corresponding to each region/domain. Using linear modeling performed by limma package version 3.50.0, mean methylation difference and p values between the case samples and matched control samples were calculated for each probe. Blood cell proportions were estimated by the Houseman method[31] and were added to the model matrix as confounding features. The selection of probes was performed in a three-step procedure. First, for each episignature analysis, i probes with the highest product of mean methylation differences between the case and control groups and negative of the logarithm of the p values were selected, where $i = 1000, 600,$ and 900 for the ZZ, TAZ2, and ID4 cohorts. Next, j probes with the highest areas under the receiver operating curve (AUROC) were retained, where $j = 333, 300,$ and 225 for the ZZ, TAZ2, and ID4 cohorts. Finally, probes with a correlation above k, calculated within the case and control groups separately, were removed, where $k = 0.85, 0.8,$ and 0.75 for ZZ, TAZ2, and ID4. This procedure resulted in the selection of 146, 215, and 104 probes for the ZZ, TAZ2, and ID4 respectively. The values used in the three-step probe selection process are summarized in Table S1.

To assess the robustness of the selected probe sets, hierarchical clustering using Ward's method on Euclidean distance and multidimensional scaling (MDS) by scaling of the pairwise Euclidean distances between samples were performed. The assessment of the sensitivity and reproducibility of the episignatures was conducted by iterations of cross-validation, using all but one case sample for probe selection. At each trial, after selecting the set of probes using the aforementioned three-step process, an MDS plot was created to assess the clustering of the excluded case relative to the cases used for signature development in each iteration.

Binary model construction

For the ID4 cohort, the selected set of probes was used to construct a support vector machine (SVM), in order to distinguish case samples from control samples more accurately, using the e1071 package version 1.7.9, as described previously.[32,33] The classifier generates a methylation variant pathogenicity (MVP) score, ranging from 0 to 1, with higher scores indicating greater similarity to the identified episignature. The classifier was constructed by training the MKHK-ID4 case samples against the matched control samples as well as against 75% of other controls and samples from 56 other neurodevelopmental disorders (NDDs) from the previously published EpiSign v3 clinical classifier within EKD (https://episign.lhsc.on.ca/index.html).[11] The remaining 25% of these controls and other NDD samples were used for testing. Other study cohort samples were also supplied into the model to evaluate their MVP scores.

Identification of regions of differential methylation for MKHK

DMRcate package version 2.8.3[34] was used to map the differentially methylated regions (DMRs) for each protein region/domain. Regions were selected if containing a minimum of five different CpG sites within 1 kb with at least 5% mean methylation difference (with a Fisher's multiple comparison p value <0.01) between the case samples and matched control samples.

Results

Phenotype

The cohort consisted of 82 individuals (46 male) with a median age of 7 (range 0.4–48) years. All individuals had been referred to their clinician because of intellectual disability (ID) and/or behavioral problems. Table 1 summarizes common clinical and morphological characteristics per MKHK subtype, and Table 2 shows a more comprehensive list, including less common features. Photographs of the faces in frontal view are shown in Figure 1, and of the faces in lateral view, the hands, and feet in Figure S1. Table S2 lists the morphological features per subtype, and clinical and morphological characteristics per individual are listed in Table S3.

Common features that were seen in all MKHK subtypes were ID (typically mild in MKHK-ZZ, moderate to severe in MKHK-TAZ2, and mild to severe in MKHK-ID4); behavioral problems including autism spectrum disorder, cerebral anomalies, strabismus, recurrent infections, feeding problems in infancy/childhood, gastroesophageal reflux, and constipation (Tables 1 and 2). Vision and/or hearing impairments were also relatively common. Mean heights, weights, and head circumferences at birth were smaller than in the general population (Tables 1 and 2).

However, some features showed subtype specificity (Tables 1, 2, and S2). Although numbers were relatively small ($n = 9$) for individuals with the MKHK-ZZ methylation profile, a number of subtype-specific features could be discerned: overweight at last measurement (100%), hypermetropia (75%), dental anomalies (63%, mostly missing teeth), hormonal problems (50%, including thyroid disorders [$n = 3$] and growth hormone deficiency [$n = 2$] and diabetes mellitus, type II in one individual [Table S3]), and hypermobility (Tables 1 and 2). Only a few individuals had malformations, including cleft palate, congenital heart

Table 1. Summary of common characteristics of individuals with a confirmed MKHK methylation profile/episignature per MKHK subtype

	MKHK-ZZ ($n = 9$)	MKHK-TAZ2 ($n = 14$)	MKHK-ID4 ($n = 21$)
**Location of variants predicted to disturb local protein structure and/or function**			
CBP residues NP_004371.2	1,705–1,745 (ZZ domain)	1,772–1,840 (TAZ2 domain)	1,852–1,875 (first α helix of ID4)
p300 residues NP_001420.2	1,668–1,708 (ZZ domain)	1,735–1,803 (TAZ2 domain)	1,810–1,833 (first α helix of ID4)
**Common characteristics not subtype specific**			
Intellectual disability	mild 63%<br>moderate 38%	mild 8%<br>moderate 31%<br>severe 62%	mild 16%<br>moderate 53%<br>severe 21%
Behavioral problems including ASD	8 out of 8 (100%)	8 out of 12 (67%)	11 out of 18 (61%)
Last height SDS (mean ± SD)	−1.7 ± 1.2	−2.6 ± 1.1	−0.6 ± 1.7
Last weight-to-height SDS (mean ± SD)	+3.5 ± 0.8	+0.7 ± 1.3	−1.3 ± 1.9
Last OFC SDS (mean ± SD)	−1.2 ± 1.5	−2.3 ± 1.4	−1.0 ± 1.4
Strabismus	4 out of 8 (50%)	9 out of 14 (64%)	11 out of 18 (61%)
Recurrent infections	5 out of 9 (56%)	7 out of 13 (54%)	10 out of 18 (56%)
Feeding problems	5 out of 9 (56%)	13 out of 14 (93%)	18 out of 20 (90%)
Gastroesophageal reflux	2 out of 8 (25%)	7 out of 10 (70%)	7 out of 17 (41%)
Constipation	3 out of 8 (38%)	6 out of 11 (55%)	11 out of 18 (61%)
**Additional characteristics per subtype**			
Clinical characteristics (present in ≥25%)	overweight 100%<br>hypermetropia 75%<br>dental anomalies 63%<br>hormonal anomalies 50%<br>scoliosis 33%<br>hypermobility 38%	hearing impairment 45%<br>dental anomalies 42%<br>cryptorchidism 38%<br>muscle hypertrophy/hypertonia 31%<br>contractures 25%<br>anomalies of extremities 50%	hearing impairment 40%<br>cardiovascular anomaly 33%<br>renal anomaly 26%<br>cryptorchidism 33%<br>muscle hypertrophy/hypertonia 37%<br>contractures 43%<br>anomalies of extremities 39%
Morphological characteristics (present in ≥50%)	thick eyebrows 56%<br>flared eyebrows 56%<br>ptosis/blepharophimosis 50%<br>high palate 86%<br>thin vermilion upper lip 56%<br>sandal gaps 50%	thick eyebrows 54%<br>broad nasal tip 57%	prominent forehead 75%<br>sparse hair 53%<br>upslanted palpebral fissures 71%<br>short palpebral fissures 71%<br>ptosis/blepharophimosis 71%<br>protruding upper part of ears 65%<br>short nose 52%<br>depressed nasal bridge 50%<br>depressed nasal ridge 56%<br>broad nasal tip 57%<br>anteverted nares 52%<br>short columella 67%<br>high palate 59%<br>long philtrum 62%

CBP, CREB-binding protein; ID4, first α helix of the fourth intrinsically disordered region; OFC, occipito-frontal circumference; p300, E1A-associated protein; SDS, standard deviation score; TAZ2, zinc-finger TAZ-type; ZZ, zinc-finger ZZ-type. A complete list of clinical characteristics per MKHK subtype can be found in Table 2, and a complete list of morphological characteristics in Table S2. A detailed description of clinical and morphological characteristics per individual is included in Table S3.

anomaly, renal anomaly, and cryptorchidism. Other less frequent features included epilepsy, kypho/scoliosis, and hip dysplasia. Overlapping morphological features included thick and flared eyebrows, ptosis/blepharophimosis, high palate, thin vermilion of the upper lip, and sandal gaps (Figures 1 and S1).

Characteristics frequently seen in individuals with the MKHK-TAZ2 methylation profile were hearing impairment, dental anomalies, cryptorchidism, muscle hypertrophy/hypertonia, contractures, and anomalies of the extremities (mostly clubfeet). Other malformations included cleft palate, laryngeal anomaly, congenital heart anomaly, and renal anomaly. Also epilepsy, hypothyroidism, kyphosis/scoliosis, hip dysplasia, hypermobility, and inguinal hernias were seen (Tables 2 and S3). Although the individuals shared some overlapping morphological features (more than half of the individuals had thick eyebrows and a broad nasal tip), no easily recognizable facial phenotype could be discerned (Figure 1).

Almost half of the included individuals had a variant within the first α helix of ID4. Certain features were relatively more common compared to the other subtypes. As

Table 2. Clinical characteristics of individuals with a confirmed MKHK methylation profile/episignature per MKHK subtype

	MKHK-ZZ ($n = 9$)	MKHK-TAZ2 ($n = 14$)	MKHK-ID4 ($n = 21$)
**Characteristics at birth**			
Sex (male/female)	6/3 (66%/33%)	8/6 (57%/43%)	5/16 (24%/76%)
Mean gestational age (weeks)	38.8 (range 34–41)	38.6 (range 34–41)	37.8 (range 30–40)
Premature birth (<37 weeks)	1 out of 9 (11%)	2 out of 14 (14%)	3 out of 21 (14%)
Weight SDS at birth (mean ± SD)	−1.2 ± 0.8	−1.3 ± 1.3	−1.6 ± 1.0
Weight at birth <−2 SD	1 out of 9 (11%)	6 out of 14 (43%)	5 out of 21 (24%)
Length SDS at birth (mean ± SD)	−1.1 ± 1.2	−1.0 ± 0.7	−1.5 ± 1.0
Length at birth <−2 SD	1 out of 9 (11%)	1 out of 7 (14%)	5 out of 14 (36%)
OFC SDS at birth (mean ± SD)	−1.7 ± 0.9	−1.3 ± 1.0	−0.1 ± 2.9
OFC at birth < −2 SD	3 out of 9 (33%)	1 out of 10 (10%)	1 out of 11 (9%)
**Growth parameters at last measurement**			
Last height SDS (mean ± SD)	−1.7 ± 1.2	−2.6 ± 1.1	−0.6 ± 1.7
Height last <−2 SD	2 out of 9 (22%)	6 out of 14 (43%)	3 out of 18 (17%)
Last weight-to-height SDS (mean ± SD)	+3.5 ± 0.8	+0.7 ± 1.3	−1.3 ± 1.9
Weight to height last <−2 SD	0	0 out of 14	3 out of 17 (18%)
Weight to height last > +2 SD	7 out of 7 (100%)	2 out of 14 (14%)	0 out of 17
Last OFC SDS (mean ± SD)	−1.2 ± 1.5	−2.3 ± 1.4	−1.0 ± 1.4
OFC last <−2 SD	3 out of 8 (38%)	7 out of 12 (58%)	4 out of 18 (22%)
**Development and behavior**			
ID	8 out of 8 (100%)	13 out of 13 (100%)	18 out of 19 (95%)
Mild ID	5 out of 8 (63%)	1 out of 13 (8%)	3 out of 19 (16%)
Moderate ID	3 out of 8 (38%)	4 out of 13 (31%)	10 out of 19 (53%)
Severe ID	0	8 out of 13 (62%)	4 out of 19 (21%)
Age at first walking in years (mean ± SD)	1.4 ± 0.4	2.9 ± 1.7 (not yet $n = 4$; 1.5–5.9 years)	2.4 ± 1.4 (not yet $n = 2$; 3.8 years)
Age at first words in years (mean ± SD)	2.3 ± 1.2	2.3 ± 0.6 (not yet $n = 9$; 1.5–24 years)	3.3 ± 1.7 (not yet $n = 5$; 3.8–38 years)
Behavioral problems (including ASD)	8 out of 8 (100%)	8 out of 12 (67%)	11 out of 18 (61%)
Autism spectrum disorder/autistic behavior	4 out of 8 (50%)	4 out of 12 (33%)	7 out of 18 (39%)
**Senses**			
Hypermetropia	6 out of 8 (75%)	3 out of 14 (21%)	4 out of 18 (22%)
Myopia	1 out of 8 (13%)	4 out of 14 (29%)	3 out of 18 (17%)
Strabismus	4 out of 8 (50%)	9 out of 14 (64%)	11 out of 18 (61%)
Other visual impairments	4 out of 9 (44%)	4 out of 14 (29%)	8 out of 20 (40%)
Hearing impairment	2 out of 9 (22%)	5 out of 11 (45%)	8 out of 20 (40%)
**Neurological and endocrinal**			
Epilepsy	1 out of 9 (11%)	3 out of 14 (21%)	3 out of 20 (15%)
Hormonal anomalies	4 out of 8 (50%)	1 out of 11 (9%)	1 out of 17 (6%)
**Ear, nose, throat**			
Recurrent infections	5 out of 9 (56%)	7 out of 13 (54%)	10 out of 18 (56%)
Cleft palate	1 out of 9 (11%)	1 out of 13 (8%)	1 out of 20 (5%)

(*Continued on next page*)

Table 2. *Continued*

	MKHK-ZZ ($n = 9$)	MKHK-TAZ2 ($n = 14$)	MKHK-ID4 ($n = 21$)
Dental anomalies	5 out of 8 (63%)	5 out of 12 (42%)	3 out of 15 (20%)
Laryngeal anomaly	0 out of 7	1 out of 12 (8%)	4 out of 17 (24%)
Problems with intubation	0 out of 8	0 out of 9	2 out of 16 (13%)
**Cardiovascular**			
Cardiovascular anomaly	2 out of 9 (22%)	2 out of 12 (17%)	7 out of 21 (33%)
**Gastroenterological**			
Feeding problems in infancy/childhood	5 out of 9 (56%)	13 out of 14 (93%)	18 out of 20 (90%)
Gastroesophageal reflux	2 out of 8 (25%)	7 out of 10 (70%)	7 out of 17 (41%)
Constipation	3 out of 8 (38%)	6 out of 11 (55%)	11 out of 18 (61%)
Gastrointestinal anomaly	0 out of 9	0 out of 13	2 out of 18 (11%)
**Renal and genitourinary**			
Renal anomaly	2 out of 9 (22%)	2 out of 12 (17%)	5 out of 19 (26%)
Cryptorchidism	1 out of 6 males (17%)	3 out of 8 males (38%)	2 out of 6 males (33%)
**Musculoskeletal**			
Scoliosis	3 out of 9 (33%)	1 out of 12 (8%)	4 out of 20 (20%)
Kyphosis	1 out of 9 (11%)	1 out of 12 (8%)	2 out of 20 (10%)
Umbilical hernia	0 out of 9	0 out of 12	2 out of 19 (10%)
Inguinal hernia	0 out of 9	2 out of 12 (17%)	4 out of 19 (21%)
Hip dysplasia	2 out of 9 (22%)	2 out of 12 (17%)	3 out of 19 (16%)
Hypermobility	3 out of 8 (38%)	1 out of 11 (9%)	3 out of 17 (18%)
Muscle hypertrophy/hypertonia	1 out of 9 (11%)	4 out of 13 (31%)	7 out of 19 (37%)
Contractures	0 out of 9	3 out of 12 (25%)	9 out of 21 (43%)
Anomalies of the extremities	1 out of 9 (11%)	7 out of 14 (50%)	7 out of 18 (39%)

ASD, autism spectrum disorder; ID, intellectual disability; OFC, occipito-frontal circumference; SDS, standard deviation score. A more detailed description of clinical characteristics in each of the individuals is included in Table S3.

in MKHK-TAZ2, hearing impairment, cryptorchidism, muscle hypertrophy/hypertonia, contractures, and anomalies of the extremities (mostly clubfeet) were relatively frequently seen. Apart from these, cardiovascular and renal anomalies were also seen in more than a quarter of the individuals. Other malformations included cleft palate, laryngeal anomaly, and gastrointestinal malformations (esophageal segment stenosis $n = 1$, malrotation and rectovaginal fistula $n = 1$). A range of other characteristics were less often seen (epilepsy, inguinal and abdominal hernia, kyphosis/scoliosis, joint hypermobility, hip dysplasia, and growth hormone deficiency; Tables 2 and S3). Notably, problems with intubation were reported twice. The individuals with the MKHK-ID4 subtype had a recognizable phenotype consisting of several features that are seen in more than half of the individuals, including a prominent forehead, short and upslanted palpebral fissures, ptosis/blepharophimosis, protruding upper part of the ears, depressed nasal bridge and ridge, short nose, broad nasal tip, short columella, anteverted nares, and long philtrum (Tables 1 and S2). Individuals with the recurrent variant c.5602C>T, p.(Arg1868Trp) ($n = 14$) presented with severe ID more frequently compared to the other individuals with MKHK-ID4 (Table S3).

Genotype

In all individuals for whom no clinical diagnosis was suggested, molecular analysis had been performed either by using a panel targeted for genes known to be mutated in individuals with ID or by untargeted exome or genome sequencing. Directed Sanger sequencing of *CREBBP* had been performed because of the suspicion of a mild form of RSTS in individual C.T.10, because of clinical suspicion of MKHK in C.I.22 and C.I.33, and because of an affected parent in C.T.22. The sequencing method for individual C.I.14 was unknown. Seventy-one individuals had a variant in *CREBBP*, and 11 individuals had variants in *EP300*. Thirteen had a variant in the ZZ domain, two individuals had variants between the ZZ and TAZ2 domains, 27 individuals in the TAZ2 domain, one individual in between TAZ2 and ID4, and 39 individuals in ID4 (Figure 2).

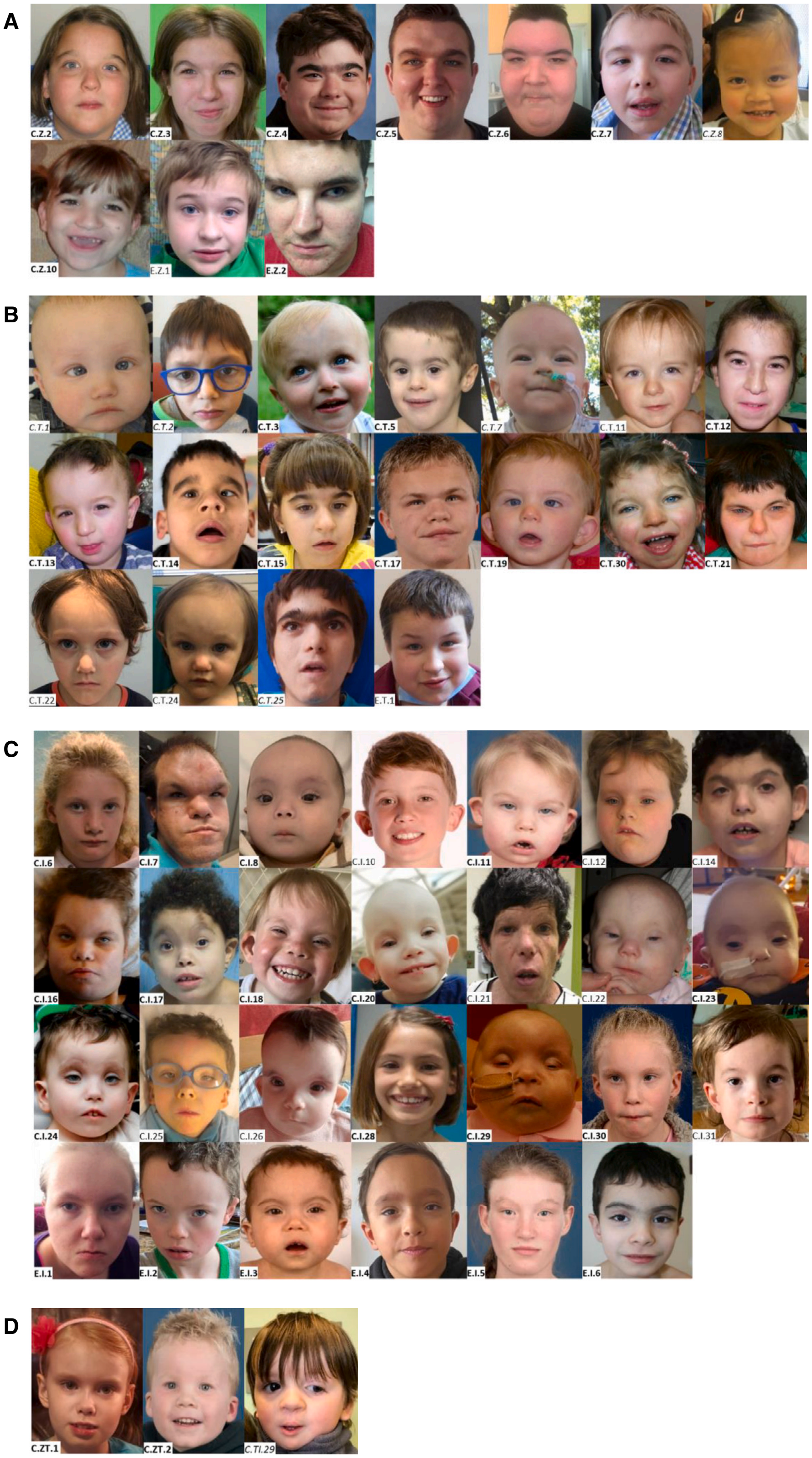

Figure 1. Facial morphology of the presently described individuals with a variant in the MKHK region of *CREBBP* and *EP300*
Facial features of individuals with a variant in (A) the ZZ domain, (B) TAZ2-domain, (C) ID4, and (D) in between the ZZ and TAZ2-domain (C.ZT.1 en C.ZT.2) and in between TAZ2 and ID4 (C.TI.29). Study numbers are depicted within each photograph reflecting the affected gene (C or E representing *CREBBP* and *EP300*) and domain (Z, T, I, ZT, and TI representing ZZ, TAZ2, ID4, the region between ZZ and TAZ2, and the region between TAZ2 and ID4, respectively) followed by a unique number. Study numbers of individuals with a confirmed MKHK episignature or methylation profile are displayed in bold, and those of individuals who were tested but in whom no MKHK episignature or methylation profile was found are in italics. Photographs of the lateral facial characteristics and of the hands and feet are shown in Figure S1. Detailed description of facial and distal limb morphology of all individuals can be found in Table S3.

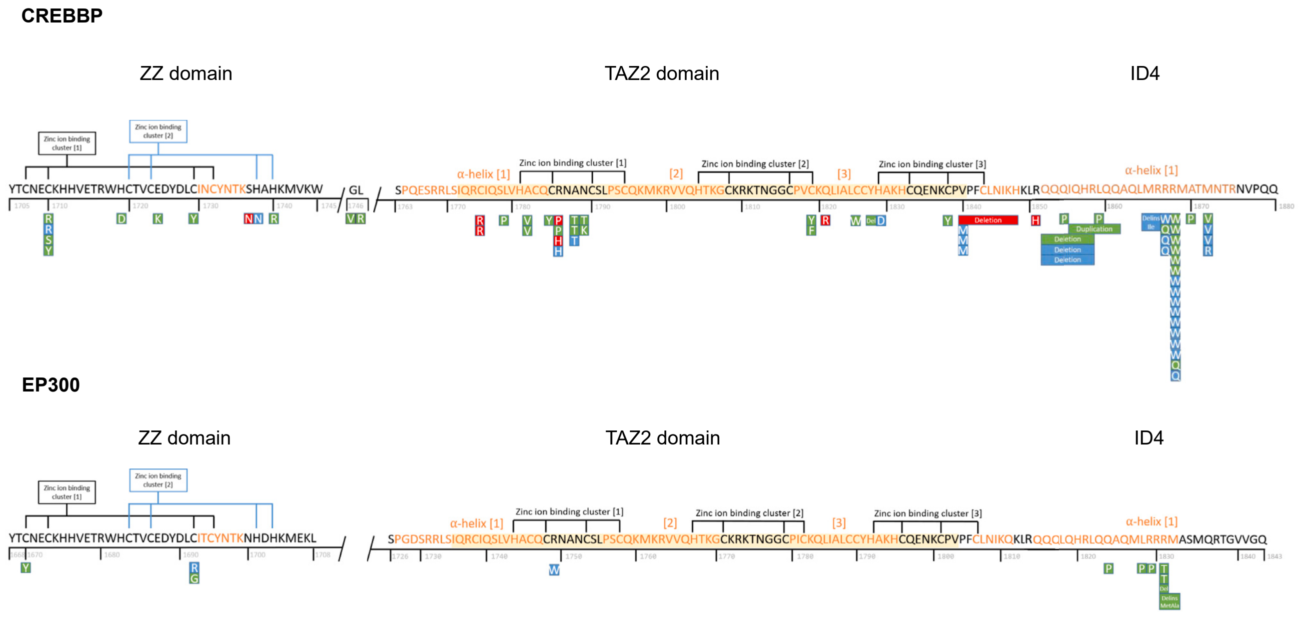

Figure 2. Schematic overview of the predicted amino acid changes within the MKHK region of CBP (NP_004371.2) and p300 (NP_001420.2)
ZZ, zinc-finger ZZ-type (CBP residues 1,705–1,745; p300 1,668–1,708); TAZ2, zinc-finger TAZ-type (CBP 1,772–1,840; p300 1,735–1,803); ID4, first α helix of the fourth intrinsically disordered region (CBP residues 1,852–1,875, p300 1,810–1,833). Yellow shaded residues in TAZ2 as defined in NCBI reference. Note that three variants are located outside these domains/region (two in between ZZ and TAZ2 and one in between TAZ2 and ID4). Green box represents confirmed methylation profile or episignature (in ID4). Red box, no methylation profile or episignature; blue, no result available. The ID4 episignature was also found in the two individuals with a variant in between ZZ and TAZ2 (C.ZT.1 and moderately in C.ZT.2).

The variants, predicted protein changes, variant inheritance, episignature/methylation profile results, and ACMG criteria are shown in Table 3. This table is extended with *in silico* prediction scores in Table S4.

AlphaFold protein structures of CBP/p300 are shown in Figure 3, with variants mostly clustering around zinc-ion-binding residues in the ZZ and TAZ2 domains, and around residues in ID4 that form hydrogen bonds with the HAT domain.

DNA methylation analysis

DNA for methylation analysis was available for 54 individuals (27 males), of whom 10 had a variant in the ZZ domain, 20 in the TAZ2 domain, and 21 in ID4. The remaining three individuals had a variant located between ZZ and TAZ2 ($n = 2$) and between TAZ2 and ID4 ($n = 1$).

For the ID4 region in CBP/p300, a highly sensitive and specific episignature was refined (21 out of 21 individuals). While a milder methylation profile was detected for the ZZ (found in nine out of 10 tested individuals) and TAZ2 (in 14 out of 20) domains, these profiles did not meet the stringent criteria of an episignature clinical biomarker at this time. In all individuals who were tested but did not show any of the MKHK methylation profiles, no RSTS episignature, nor any of the other known episignatures, was found (data not shown). Domain-specific methylation profiles were discerned for the ZZ domain in CBP/p300 and TAZ2 domain in CBP, while a domain-specific diagnostic episignature was refined for the ID4 domain in CBP/p300 (in 21 out of 21).

The ZZ domain-associated methylation profile

Ten samples from individuals with variants in the ZZ domain of CBP/p300 were used for the analysis. One of them (C.Z.8) did not present a methylation profile similar to the rest of the cohort, and, hence, was excluded from the discovery cohort and included in the testing cohort. Fifty-four control samples were selected and matched to the nine ZZ samples by age, sex, and array type. The 146 selected probes were used to perform hierarchical clustering and MDS models (Figures 4A and 4B). These plots indicated that the selected set of probes were robust in differentiating between the case and control groups. Nine rounds of cross-validation were performed, using eight ZZ samples (leaving one out) and the 54 matched controls for probe selection at each round. An MDS model was then performed for all trials. In three out of nine rounds, the testing sample clustered with control samples and, in two other rounds, the testing sample fell between case and control groups (Figure 4C). This indicated that the identified probe set, while demonstrating mild subtype-specific methylation differences, is currently not yet sufficiently reproducible to meet the stringent criteria of a sensitive diagnostic episignature with the current dataset. The term mild methylation profile thus refers to not being robust enough to use as a diagnostic tool, which we expect to be the case once more samples will be included.

The TAZ2 domain-associated methylation profile

There were 20 individuals with variants in the TAZ2 domain for whom samples were available. To map the

Table 3. Genotypes, methylation profiles/episignatures, and ACMG criteria for the variants in *CREBBP* (GenBank: NM_004380.2) or *EP300* (GenBank: NM_001429.3)

	Gene	Domain	DNA variant	Predicted protein change	Inheritance	Episignature	ACMG before DNA methylation	ACMG after DNA methylation
C.Z.1	*CREBBP*	ZZ	c.5128T>C	p.(Cys1710Arg)	*de novo*	N/A	likely pathogenic	–
C.Z.2	*CREBBP*	ZZ	c.5128T>A	p.(Cys1710Ser)	*de novo*	MKHK_ZZ	likely pathogenic	pathogenic
C.Z.3	*CREBBP*	ZZ	c.5128T>C	p.(Cys1710Arg)	*de novo*	MKHK_ZZ	likely pathogenic	pathogenic
C.Z.4	*CREBBP*	ZZ	c.5129G>A	p.(Cys1710Tyr)	*de novo*	MKHK_ZZ	likely pathogenic	pathogenic
C.Z.5	*CREBBP*	ZZ	c.5155C>G	p.(His1719Asp)	*de novo*	MKHK_ZZ	likely pathogenic	pathogenic
C.Z.6	*CREBBP*	ZZ	c.5170G>A	p.(Glu1724Lys)	*de novo*	MKHK_ZZ	likely pathogenic	pathogenic
C.Z.7	*CREBBP*	ZZ	c.5186G>A	p.(Cys1729Tyr)	*de novo*	MKHK_ZZ	likely pathogenic	pathogenic
C.Z.8	*CREBBP*	ZZ	c.5210G>A	p.(Ser1737Asn)	unknown	None	VUS	–
C.Z.9	*CREBBP*	ZZ	c.5212C>A	p.(His1738Asn)	unknown	N/A	VUS	–
C.Z.10	*CREBBP*	ZZ	c.5219A>G	p.(His1740Arg)	*de novo*	MKHK_ZZ	likely pathogenic	pathogenic
E.Z.3	*EP300*	ZZ	c.5009G>A	p.(Cys1670Tyr)	*de novo*	MKHK_ZZ	likely pathogenic	pathogenic
E.Z.1	*EP300*	ZZ	c.5074T>C	p.(Cys1692Arg)	*de novo*	N/A	likely pathogenic	–
E.Z.2	*EP300*	ZZ	c.5074T>G	p.(Cys1692Gly)	*de novo*	MKHK_ZZ	likely pathogenic	pathogenic
C.ZT.1	*CREBBP*	In between ZZ and TAZ2	c.5237G>T	p.(Gly1746Val)	*de novo*	MKHK_ID4	VUS	likely pathogenic
C.ZT.2	*CREBBP*	In between ZZ and TAZ2	c.5240T>G	p.(Leu1747Arg)	*de novo*	Moderate MKHK_ID4	VUS	likely pathogenic
C.T.1	*CREBBP*	TAZ2	c.5323T>C	p.(Cys1775Arg)	maternally inherited	None	likely pathogenic	–
C.T.2	*CREBBP*	TAZ2	c.5323T>C	p.(Cys1775Arg)	*de novo*	None	likely pathogenic	–
C.T.3	*CREBBP*	TAZ2	c.5336T>C	p.(Leu1779Pro)	*de novo*	MKHK_TAZ2	likely pathogenic	pathogenic
C.T.4	*CREBBP*	TAZ2	c.5345C>T	p.(Ala1782Val)	*de novo*	MKHK_TAZ2	likely pathogenic	pathogenic
C.T.5	*CREBBP*	TAZ2	c.5345C>T	p.(Ala1782Val)	*de novo*	MKHK_TAZ2	likely pathogenic	pathogenic
C.T.6	*CREBBP*	TAZ2	c.5354G>A	p.(Cys1785Tyr)	*de novo*	MKHK_TAZ2	likely pathogenic	pathogenic
C.T.8	*CREBBP*	TAZ2	c.5357G>A	p.(Arg1786His)	*de novo*	None	likely pathogenic	–
C.T.9	*CREBBP*	TAZ2	c.5357G>A	p.(Arg1786His)	*de novo*	N/A	likely pathogenic	–
C.T.7[a]	*CREBBP*	TAZ2	c.5357G>C	p.(Arg1786Pro)	*de novo*	None	likely pathogenic	–
C.T.10	*CREBBP*	TAZ2	c.5357G>C	p.(Arg1786Pro)	*de novo*	MKHK_TAZ2	likely pathogenic	pathogenic
C.T.11	*CREBBP*	TAZ2	c.5362G>A	p.(Ala1788Thr)	*de novo*	N/A	pathogenic	–
C.T.12[b]	*CREBBP*	TAZ2	c.5362G>A	p.(Ala1788Thr)	*de novo*	MKHK_TAZ2	pathogenic	pathogenic
C.T.13[b]	*CREBBP*	TAZ2	c.5362G>A	p.(Ala1788Thr)	*de novo*	MKHK_TAZ2	pathogenic	pathogenic
C.T.14	*CREBBP*	TAZ2	c.5366A>C	p.(Asn1789Thr)	*de novo*	MKHK_TAZ2	likely pathogenic	pathogenic
C.T.15	*CREBBP*	TAZ2	c.5367C>G	p.(Asn1789Lys)	*de novo*	MKHK_TAZ2	likely pathogenic	pathogenic
C.T.16	*CREBBP*	TAZ2	c.5456G>A	p.(Cys1819Tyr)	*de novo*	MKHK_TAZ2	likely pathogenic	pathogenic
C.T.17	*CREBBP*	TAZ2	c.5456G>T	p.(Cys1819Phe)	*de novo*	MKHK_TAZ2	likely pathogenic	pathogenic
C.T.18	*CREBBP*	TAZ2	c.5462A>G	p.(Gln1821Arg)	*de novo*	None	likely pathogenic	–
C.T.19	*CREBBP*	TAZ2	c.5478C>G	p.(Cys1826Trp)	*de novo*	MKHK_TAZ2	likely pathogenic	pathogenic
C.T.30	*CREBBP*	TAZ2	c.5482_5484del	p.(Tyr1828del)	*de novo*	MKHK_TAZ2	likely pathogenic	pathogenic
C.T.20	*CREBBP*	TAZ2	c.5485C>G	p.(His1829Asp)	*de novo*	N/A	likely pathogenic	–
C.T.21	*CREBBP*	TAZ2	c.5513G>A	p.(Cys1838Tyr)	*de novo*	MKHK_TAZ2	likely pathogenic	pathogenic
C.T.22[b]	*CREBBP*	TAZ2	c.5518G>A	p.(Val1840Met)	paternally inherited	N/A	VUS	–

(*Continued on next page*)

Table 3. *Continued*

	Gene	Domain	DNA variant	Predicted protein change	Inheritance	Episignature	ACMG before DNA methylation	ACMG after DNA methylation
C.T.23[b]	*CREBBP*	TAZ2	c.5518G>A	p.(Val1840Met)	unknown	N/A	VUS	–
C.T.24[b]	*CREBBP*	TAZ2	c.5518G>A	p.(Val1840Met)	paternally inherited	N/A	VUS	–
C.T.25[a]	*CREBBP*	TAZ2	c.5518_5544del	p.(Val1840_His1848del)	*de novo*	None	likely pathogenic	–
E.T.1	*EP300*	TAZ2	c.5245C>T	p.(Arg1749Trp)	*de novo*	N/A	likely pathogenic	–
C.TI.29[a]	*CREBBP*	in between TAZ2 and ID4	c.5552G>A	p.(Arg1851His)	*de novo*	none	VUS	–
C.I.3[b]	*CREBBP*	ID4	c.5555_5575del	p.(Gln1852_Arg1858del)	*de novo*	MKHK_ID4	pathogenic	pathogenic
C.I.4[b]	*CREBBP*	ID4	c.5555_5575del	p.(Gln1852_Arg1858del)	*de novo*	N/A	pathogenic	–
C.I.5[b]	*CREBBP*	ID4	c.5555_5575del	p.(Gln1852_Arg1858del)	*de novo*	N/A	pathogenic	–
C.I.6	*CREBBP*	ID4	c.5561A>C	p.(Gln1854Pro)	*de novo*	MKHK_ID4	likely pathogenic	pathogenic
C.I.7	*CREBBP*	ID4	c.5563_5583dup	p.(Ile1855_ Gln1861dup)	*de novo*	MKHK_ID4	likely pathogenic	pathogenic
C.I.8	*CREBBP*	ID4	c.5576T>C	p.(Leu1859Pro)	*de novo*	MKHK_ID4	likely pathogenic	pathogenic
C.I.9	*CREBBP*	ID4	c.5595_5597del	p.(Met1865_ Arg1866delinsIle)	*de novo*	N/A	likely pathogenic	–
C.I.10	*CREBBP*	ID4	c.5599C>T	p.(Arg1867Trp)	*de novo*	N/A	likely pathogenic	–
C.I.11	*CREBBP*	ID4	c.5600G>A	p.(Arg1867Gln)	*de novo*	MKHK_ID4	likely pathogenic	pathogenic
C.I.12[a]	*CREBBP*	ID4	c.5600G>A	p.(Arg1867Gln)	*de novo*	N/A	likely pathogenic	–
C.I.13[a]	*CREBBP*	ID4	c.5600G>A	p.(Arg1867Gln)	unknown	N/A	likely pathogenic	–
C.I.14	*CREBBP*	ID4	c.5602C>T	p.(Arg1868Trp)	*de novo*	N/A	pathogenic	–
C.I.15	*CREBBP*	ID4	c.5602C>T	p.(Arg1868Trp)	*de novo*	N/A	pathogenic	–
C.I.16	*CREBBP*	ID4	c.5602C>T	p.(Arg1868Trp)	*de novo*	MKHK_ID4	pathogenic	pathogenic
C.I.17	*CREBBP*	ID4	c.5602C>T	p.(Arg1868Trp)	*de novo*	MKHK_ID4	pathogenic	pathogenic
C.I.18	*CREBBP*	ID4	c.5602C>T	p.(Arg1868Trp)	*de novo*	MKHK_ID4	pathogenic	pathogenic
C.I.19	*CREBBP*	ID4	c.5602C>T	p.(Arg1868Trp)	*de novo*	N/A	pathogenic	–
C.I.20	*CREBBP*	ID4	c.5602C>T	p.(Arg1868Trp)	*de novo*	MKHK_ID4	pathogenic	pathogenic
C.I.21	*CREBBP*	ID4	c.5602C>T	p.(Arg1868Trp)	*de novo*	N/A	pathogenic	–
C.I.22	*CREBBP*	ID4	c.5602C>T	p.(Arg1868Trp)	unknown	N/A	pathogenic	–
C.I.23	*CREBBP*	ID4	c.5602C>T	p.(Arg1868Trp)	*de novo*	MKHK_ID4	pathogenic	pathogenic
C.I.24	*CREBBP*	ID4	c.5602C>T	p.(Arg1868Trp)	*de novo*	MKHK_ID4	pathogenic	pathogenic
C.I.25	*CREBBP*	ID4	c.5602C>T	p.(Arg1868Trp)	*de novo*	N/A	pathogenic	–
C.I.26	*CREBBP*	ID4	c.5602C>T	p.(Arg1868Trp)	*de novo*	N/A	pathogenic	–
C.I.34	*CREBBP*	ID4	c.5602C>T	p.(Arg1868Trp)	*de novo*	N/A	pathogenic	–
C.I.27	*CREBBP*	ID4	c.5603G>A	p.(Arg1868Gln)	*de novo*	N/A	pathogenic	–
C.I.28	*CREBBP*	ID4	c.5603G>A	p.(Arg1868Gln)	*de novo*	MKHK_ID4	pathogenic	pathogenic
C.I.29	*CREBBP*	ID4	c.5608G>C	p.(Ala1870Pro)	*de novo*	MKHK_ID4	likely pathogenic	pathogenic
C.I.30	*CREBBP*	ID4	c.5614A>G	p.(Met1872Val)	*de novo*	MKHK_ID4	pathogenic	pathogenic
C.I.31	*CREBBP*	ID4	c.5614A>G	p.(Met1872Val)	*de novo*	N/A	pathogenic	–
C.I.32	*CREBBP*	ID4	c.5614A>G	p.(Met1872Val)	*de novo*	N/A	pathogenic	–
C.I.33	*CREBBP*	ID4	c.5615T>G	p.(Met1872Arg)	*de novo*	N/A	likely pathogenic	–
E.I.1	*EP300*	ID4	c.5471A>C	p.(Gln1824Pro)	*de novo*	MKHK_ID4	likely pathogenic	pathogenic

(*Continued on next page*)

Table 3. *Continued*

	Gene	Domain	DNA variant	Predicted protein change	Inheritance	Episignature	ACMG before DNA methylation	ACMG after DNA methylation
E.I.2	*EP300*	ID4	c.5483T>C	p.(Leu1828Pro)	*de novo*	MKHK_ID4	likely pathogenic	pathogenic
E.I.3	*EP300*	ID4	c.5486G>C	p.(Arg1829Pro)	*de novo*	MKHK_ID4	likely pathogenic	pathogenic
E.I.5	*EP300*	ID4	c.5492_5494del	p.(Arg1831del)	*de novo*	MKHK_ID4	likely pathogenic	pathogenic
E.I.4	*EP300*	ID4	c.5492_ 5495delinsTGGC	p.(Arg1831_ Met1832delinsMetAla)	unknown	MKHK_ID4	VUS	likely pathogenic
E.I.6	*EP300*	ID4	c.5492G>C	p.(Arg1831Thr)	*de novo*	MKHK_ID4	likely pathogenic	pathogenic
E.I.7	*EP300*	ID4	c.5492G>C	p.(Arg1831Thr)	unknown	MKHK_ID4	likely pathogenic	pathogenic

N/A, not applicable; VUS, variant of uncertain significance.
[a]Additional variants were found in the following individuals: C.T.7, *CREBBP* (NM_004380.2): c.7105C>A, p.(Pro2369Thr), de novo, VUS; C.T.25, GRCh37: del10q21.3 65.73-66.26 Mb (containing no genes), maternally inherited, VUS; C.TI.29, *MED12* (NM_005120.3), p.(Gly2141Glu), maternally inherited, VUS; C.I.12, GRCh 37: del6p12.3 392 kb (including *RUNX2*), de novo, pathogenic; and C.I.13, GRCh 37: dup9q34.3 (140,722,407-141,020,389)x3 (including *CACNA1B* & *EHMT1*), inheritance unknown, VUS.
[b]C.T.12 and C.T.13 are siblings; C.T.22 and C.T.24 are siblings and children of C.T.23, who is their father; C.I.3, C.I.4, and C.I.5 are siblings.

methylation profile of the TAZ2 samples, 60 controls were selected by matching to the 20 case samples by sex, age, and array type to define the 215 episignature probes. Hierarchical clustering and MDS demonstrated a mild methylation difference between the TAZ2 and matched control individuals using the selected set of probes (Figures 5A and 5B). The sensitivity of the methylation was assessed by 20 iterations of leave-one-out cross-validation, using 19 out of 20 TAZ2 samples for probe selection at each round. In six out of 20 rounds, the testing sample clustered with control samples and in five out of 20 rounds the testing sample fell between the case and control samples (Figure 5C). Therefore, while showing overall a mild methylation profile, the selected set of probes did not demonstrate sufficient sensitivity to meet the criteria for a diagnostic episignature classifier at this time. As in MKHK-ZZ, the methylation profile was currently not robust enough to use as a diagnostic tool, which we expect to be the case once more samples are included.

The first α helix of ID4-associated episignature

In this study, the existing MKHK-ID4 episignature was refined by including eight additional samples. Sixty-three control samples were matched to the 21 previously and presently published individuals by age, sex, and array type and 104 episignature probes were selected. To assess the robustness of the selected probes in differentiating between the case and control groups, those probes were used to perform hierarchical clustering and MDS models (Figures 6A and 6B). The sensitivity and reproducibility of the episignature was verified by performing 21 iterations of cross-validation, leaving one ID4 case sample out at each trial, selecting the probes using the remaining 20 ID4 case samples and the 63 matched controls, and performing MDS using the selected set of probes to examine whether the case sample that was not used for probe selection would cluster with the remaining ID4 case samples. In all rounds, the testing sample clustered with the training case samples, and outside of the control cluster and the MKHK-ZZ and MKHK-TAZ2 group, demonstrating evidence of a robust episignature classifier (Figure 6C).

Using the selected probes for the ID4 episignature, an SVM classifier was constructed. Other MKHK samples were also supplied to the classifier and only individuals C.ZT.1 and C.ZT.2 received high MVP scores, indicating that the methylation profiles were highly similar to the ID4 episignature (Figure 6D).

Detection of the DMRs

For individuals with variants in each protein domain/region, the existence of DMRs was investigated. The selection criteria for DMRs were to contain at least five CpGs within 1 kb with a minimum mean methylation difference of 5% between the case and control groups, and a Fisher's multiple comparison p value below 0.01. For individuals with variants in the ID4 region, 74 DMRs were detected (Table S5), while those with variants in the ZZ and TAZ2 domains did not demonstrate any DMRs. Relevance of these DMRs for disease pathology will be investigated in a follow-up study by our group.

Discussion

By evaluating a cohort of 81 individuals with variants within the MKHK region of *EP300* or *CREBBP*, this study showed that MKHK consists of at least three subtypes: MKHK-ZZ, MKHK-TAZ2, and MKHK-ID4. Most variants clustered around the zinc-ion-binding residues in the ZZ and TAZ2 domains and within the first α helix of ID4 of CBP and p300. By evaluating morphological and physical characteristics, three phenotypes were discerned, corresponding to each of these domains/regions. These findings were further substantiated by the discovery of domain-specific (currently mild, yet distinct) methylation profiles for MKHK-ZZ and MKHK-TAZ2, as well as by the refinement of the previously described MKHK-ID4 episignature. Although OMIM currently categorizes MKHK into type 1

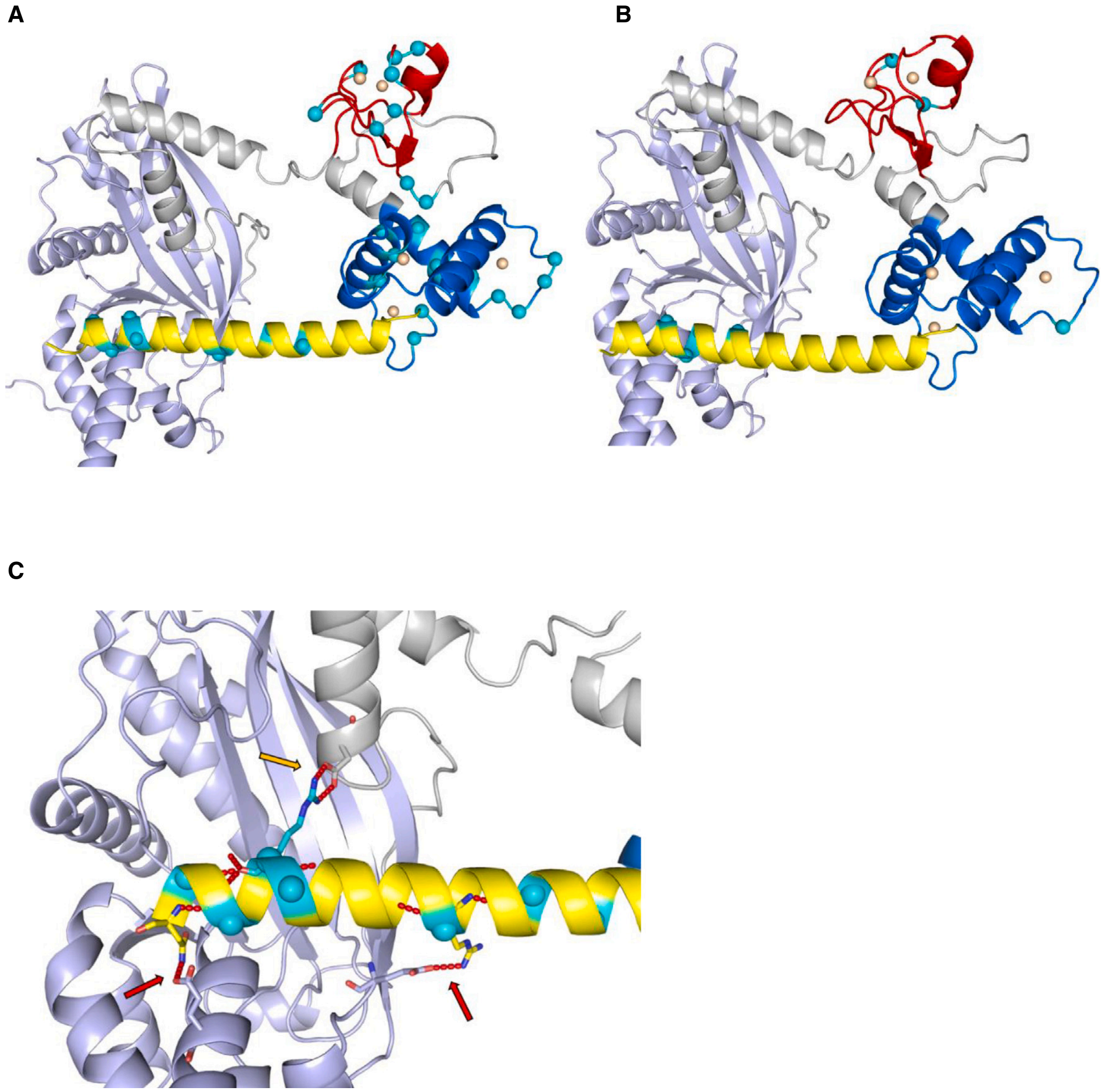

Figure 3. 3D predicted protein structures of CBP (NP_004371.2) and p300 (NP_001420.2)

(A) 3D predicted protein structure of CBP, including HAT domain (1,342–1,649, in purple), ZZ domain (1,705–1,745, in red), TAZ2-domain (1,772–1,840, in blue) and first α helix of ID4 (1,852–1,875, in yellow). Gray structures not part of functional domain according to NCBI consensus (CDD:239077 and CDD:426615). Cyan spheres represent residue variants, duplications and deletions not included. Orange spheres represent zinc ions.

(B) 3D predicted protein structure of p300, including HAT domain (1,306–1,612), ZZ domain (1,668–1,708), TAZ2-domain (1,735–1,803), and first α helix of ID4 (1,810–1,833).

(C) ID4 to HAT domain relation in CBP. Red arrows indicate hydrogen bonds (in red) between ID4 and HAT residues: (ID4 + HAT) Arg1857 + Glu1370, Asn1873 + Glu1551. Golden arrow indicates hydrogen bond Arg1868 + Asp1665.

(OMIM: 618332) and 2 (OMIM: 618333) based on variants in *CREBBP* and *EP300*, respectively, these results suggest that a domain-specific (MKHK-ZZ, MKHK-TAZ2, MKHK-ID4) rather than gene-specific (MKHK1, MKHK2) subtypes could be discerned.

CBP and p300 are central in the regulation of transcriptional networks,[35] interacting with hundreds of transcription factors and proteins.[36] This ability originates partly from the presence of long, intrinsically disordered regions, such as ID4, between multiple CBP/P300 interaction domains.[35] Most presently reported protein alterations in CBP and p300 clustered around the zinc-ion-binding residues in the ZZ and TAZ2 domains, and within the first α helix of ID4. The ZZ and TAZ2 domains contain cysteine residues, which mediate zinc-ion coordination to stabilize helical folding and mediate interactions with numerous transcriptional regulatory proteins.[8,9] Variants in these domains were thus hypothesized to affect the coordinating properties of the two zinc-finger domains of CBP/p300 by affecting their proper folding.[6] The ID4 region has also been suggested to play a role in regulating protein function.[10] In 2018, it was hypothesized that variants in the MKHK-ID4 region, unlike the loss-of-function variants in RSTS, may result in a gain of function of CBP/p300

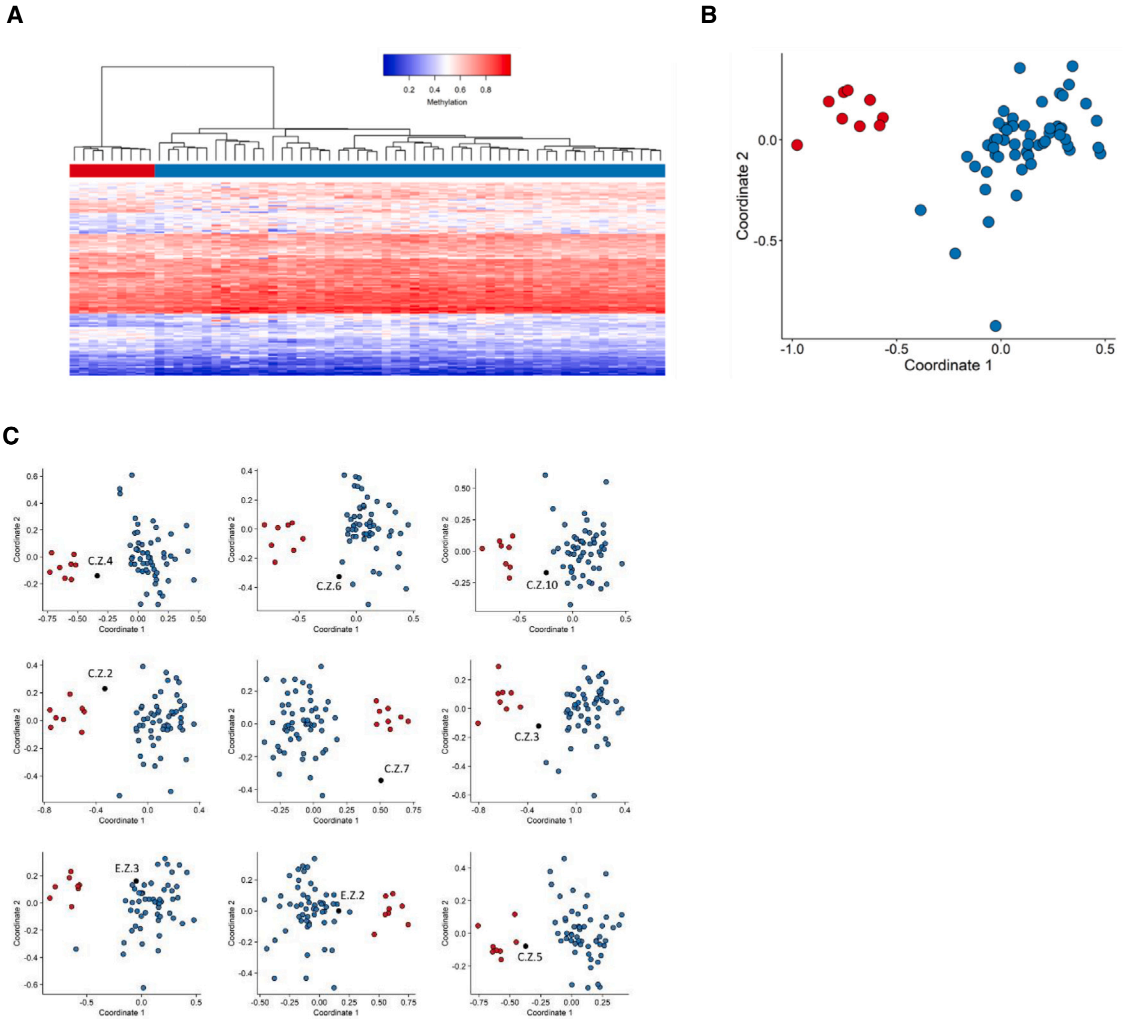

Figure 4. Assessment of the strength of the identified MKHK-ZZ methylation profile and cross-validation

Using the selected set of probes, unsupervised and supervised models were applied in order to verify the robustness, sensitivity, and specificity of the selected probes in distinguishing ZZ samples from matched control samples.

(A) Hierarchical clustering, where rows represent probes and columns represent samples. The heatmap color scale demonstrates the methylation levels from blue (no methylation) to red (full methylation). On the heatmap pane, red represents ZZ case samples and blue represents control individuals.

(B) MDS, where red and blue circles depict case and control samples, respectively. Plots A and B demonstrate clear separation of the case and control groups.

(C) In order to inspect the sensitivity of the identified ZZ methylation profile, rounds of leave-one-out cross-validation were performed, using all but one ZZ case sample for probe selection at each trial. In each MDS plot, the blue circles represent the matched control samples, red circles indicate ZZ case samples that were used for probe selection, and the black circle depicts the ZZ case sample that was left out from the probe selection process. It was observed that three ZZ samples clustered with control samples when used for testing (C.Z.6, E.Z.3, and E.Z.2) and two samples fell between case and control groups (C.Z.10 and C.Z.3), demonstrating that the selected set of probes are not sensitive enough to classify all ZZ samples correctly. Blue circles represent training samples, while gray circles depict testing samples.

proteins, since 3D facial imaging demonstrated resemblance to individuals with a duplication of 16p13.3 (the region that includes *CREBBP*),[6] and both having opposite facial features of RSTS. The findings of two recent studies on the ZZ/TAZ2/ID4 region of CBP/p300 have further substantiated this hypothesis. Sheahan et al.[37] concluded that deletion of this region is not associated with a loss of enzymatic activity but rather with modulation of CBP substrate specificity, leading to non-specific acetylation of various proteins by CBP and a potential gain of function. Ibrahim et al.[38] proposed that the first α helix of ID4 (in addition to the TAZ2 domain) of p300 plays a role in allosteric HAT regulation by displacement of the TAZ2 domain from its auto-inhibitory position, resulting in HAT activation, also indicating a possible gain of function. Our AlphaFold figures of CBP and p300 predict a close spatial proximity between the ID4 helix and HAT domain, as well as multiple hydrogen bonds between residues from each domain/region. We therefore hypothesize that the effect found in the aforementioned studies can be attributed to ID4/HAT

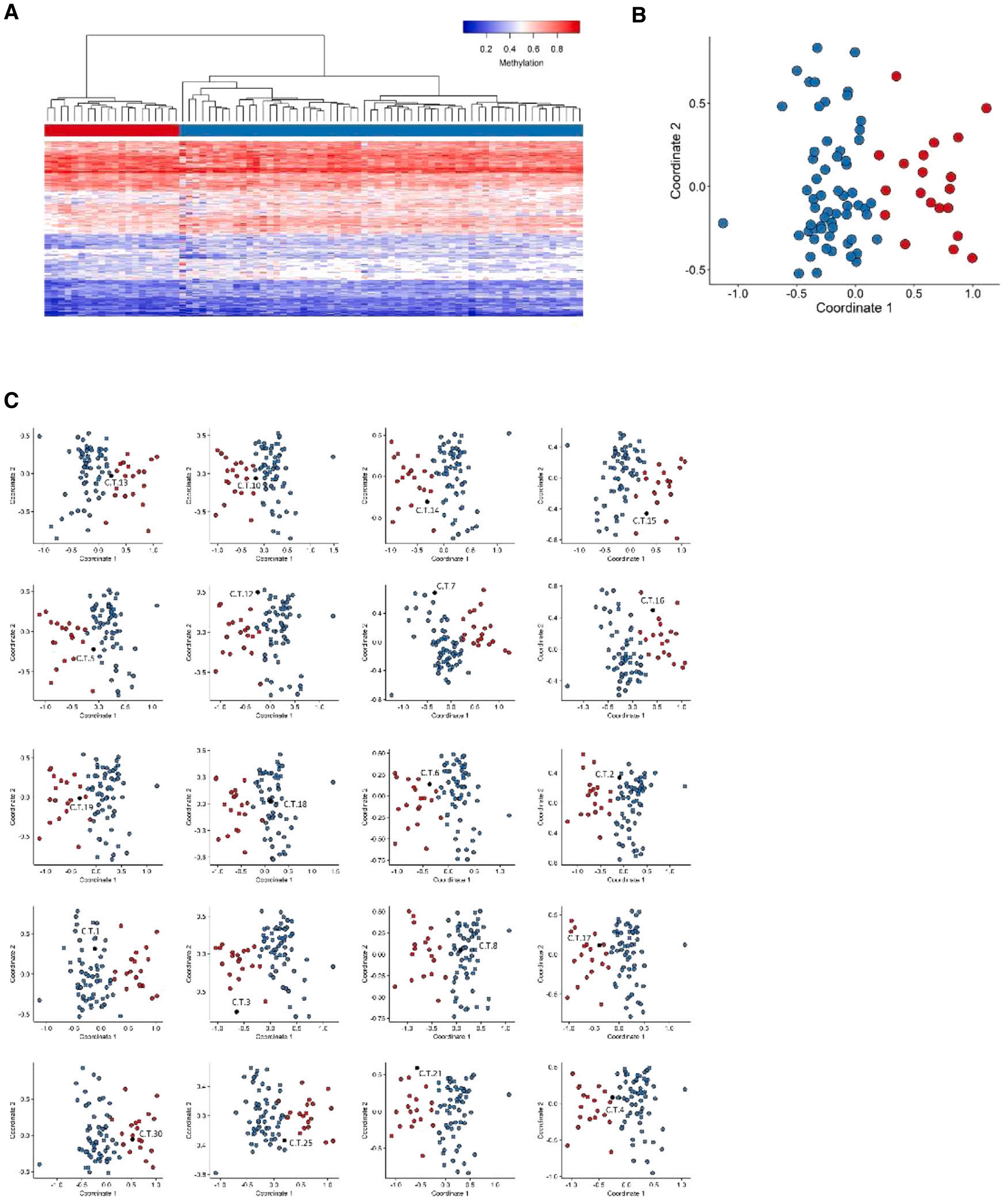

Figure 5. Assessment of the strength of the identified MKHK-TAZ2 methylation profile and cross-validation
(A) Hierarchical clustering, with red representing TAZ2 case samples and blue depicting matched control samples in the heatmap panel.
(B) MDS, where red and blue circles represent case and control samples, respectively. A mild difference is observed between the methylation patterns of the case and control groups in both plots (A and B).
(C) Cross-validation results for the TAZ2 episignature. In six iterations, the TAZ2 sample that was not used for probe selection, indicated with black, clustered with control individuals, demonstrated by blue circles (C.T.18, C.T.1, C.T.8, C.T.2, C.T.7, and C.T.25) and, in five rounds, the testing TAZ2 sample clustered between the training TAZ2 samples, red circles, and the control individuals (C.T.13, C.T.10, C.T.5, C.T.12, C.T.4), indicating that the selected probes are not sensitive enough to classify all TAZ2 samples correctly.

interaction. Variants in the ID4 helix seem to cluster around the residues that form hydrogen bonds with the HAT domain, indicating ID4/HAT interaction may indeed play an important role in MKHK-ID4 pathophysiology.

Additionally, a recent study performed by our group[39] showed that the MKHK-ID4 episignature exhibits mean global DNA hypermethylation, contrary to the RSTS1 and RSTS2 episignatures. Using the same methods,[39] preliminary evidence shows that the MKHK-ZZ and TAZ2 methylation profiles exhibited mean hypomethylation (data not shown). Functional studies are currently ongoing in our laboratory. These focus on functional impact of the presently reported

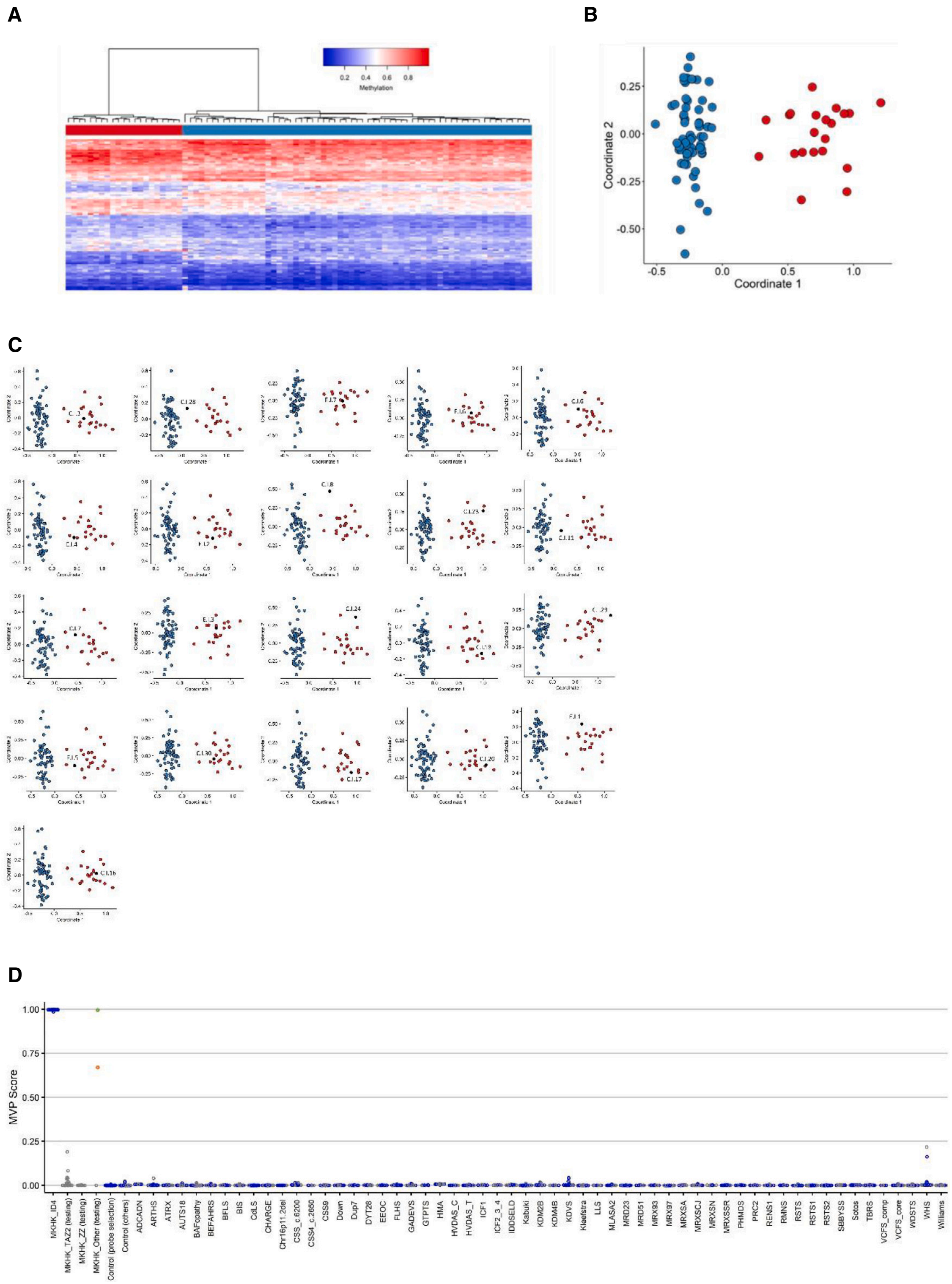

Figure 6. Assessment of the strength of the identified MKHK-ID4 episignature, cross-validation, and MVP scores
(A) Hierarchical clustering, with red representing ID4 case samples and blue depicting matched control samples in the heatmap panel.
(B) MDS, where red and blue circles represent case and control samples, respectively. Clear separation of the ID4 case samples and control samples is observed in plots A and B.
(C) Rounds of leave-one-out cross-validation were performed, using all but one ID4 case sample for probe selection at each trial. It was observed that, at all iterations, the black circle (ID4 sample not used for probe selection) clustered with the red ones (ID4 samples used for probe selection) and far from the blue circles (control individuals), demonstrating the sensitivity of the episignature.
(D) MVP scores created by the SVM constructed using the ID4 selected probes. All the ID4 case samples have received MVP scores near 1 and all the control samples and case samples from other disorders have received scores near 0, indicating full specificity of the model. All the other MKHK samples, other than individuals C.ZT.1 and C.ZT.2 (demonstrated by green and orange circle, respectively), have received low MVP scores. Blue circles represent training samples, while gray circles depict testing samples.

variants in ZZ, TAZ2, and ID4 that are needed to confirm our hypothesis that variants in ID4 result in a gain of function.

To delineate the MKHK subtypes in more detail, the characteristics of the individuals exhibiting specific MKHK methylation profiles (MKHK-ZZ, MKHK-TAZ2, MKHK-ID4) were analyzed for each subgroup (Tables 1, 2, and S3).

Although some features were common across all subtypes (e.g., ID, behavioral problems, strabismus, recurrent infections, feeding problems in infancy/childhood, gastroesophageal reflux, and constipation), some features showed somewhat more subtype specificity (Tables 1, 2, and S2). Although numbers were relatively small ($n = 9$) for individuals with the MKHK-ZZ methylation profile, this subtype was most notably marked by overweight at last measurement in all individuals, hypermetropia, dental anomalies (mostly missing teeth), and hormonal problems. Overlapping morphological features seen in about half of the individuals were thick and flared eyebrows, ptosis/blepharophimosis, high palate, thin vermilion of the upper lip, and sandal gaps (Figures 1 and S1). Individual C.Z.8 with variant p.(Ser1737Asn) who showed an atypical phenotype (e.g., an apparent flat face and a normal weight), did not show the MKHK_ZZ methylation profile. After reevaluating the conservation and *in silico* pathogenicity predictions for her variant (Table S4) in addition to the negative result of methylation analysis, this variant was classified as a variant of unknown significance (VUS) and unlikely to be pathogenic. Remarkable characteristics seen in individuals with the MKHK-TAZ2 methylation profile included muscle hypertrophy/hypertonia, contractures, and anomalies of extremities (mostly clubfeet). Although the individuals shared some overlapping morphological features (more than half of the individuals had thick eyebrows and a broad nasal tip), no easily recognizable facial phenotype could be discerned (Figure 1, Table S2).

In methylation analysis, six of the 20 tested individuals with a likely pathogenic or pathogenic variant within the TAZ2 domain clustered with control samples (C.T.1, C.T.2, C.T.7, C.T.8, C.T.18, and C.T.25) and five additional TAZ2 samples (C.T.4, C.T.5, C.T.10, C.T.12, and C.T.13) clustered between case and control groups in the leave-one-out cross-validation. Possible explanations for these individuals not (fully) showing the MKHK-TAZ2 methylation profile may be that (1) a variant had a less severe effect, e.g., p.(Arg1786His) in C.T.8 not showing the MKHK-TAZ2 methylation profile, in contrast to individual C.T.10 with variant p.(Arg1786Pro), possibly due to the effect of a change to histidine being milder than to proline[40]; (2) if case numbers increase, another methylation profile, implying another MKHK subtype, may be discerned for individuals with variants within a specific region (e.g., C.T.1 and C.T.2 with p.(Cys1775Arg) located at the end of the N-terminal region of TAZ2, and C.T.25 with a variant located at the C-terminal end of TAZ2); (3) an additional VUS, pathogenic variant, or even a polymorphism may have influenced the methylation pattern (e.g., in C.T.7 an additional VUS in *CREBBP* was found, p.(Pro2369Thr).

Almost half of the included individuals had a variant within the first α helix of ID4. Remarkable characteristics seen in individuals with MKHK-ID4 included muscle hypertrophy/hypertonia, contractures, and anomalies of extremities (mostly clubfeet), like those seen in MKHK-TAZ2. Cardiovascular and renal anomalies were seen slightly more than in both other subtypes (Tables 2 and S3). Fourteen individuals with the recurrent variant c.5602C>T, p.(Arg1868Trp) presented with severe ID more frequently compared to the other individuals with MKHK-ID4 (Table S3). The individuals with the MKHK-ID4 subtype had a recognizable phenotype consisting of a prominent forehead, short and upslanted palpebral fissures, ptosis/blepharophimosis, protruding upper part of the ears, depressed nasal bridge and ridge, short nose, broad nasal tip, short columella, anteverted nares, and long philtrum (Figures 1 and S1).

Four individuals with a variant in ID4 were previously reported by Sima et al.[14] (the recurring variant p.(Arg1868Trp)) and Nishi et al.[41] (p.(His1857_Gln1863del) and twice p.(Met1872Val)). These individuals all resembled the ID4 phenotype described here. Of note, individuals C.I.3, C.I.4, and C.I.5 were siblings, who shared the deletion p.(Gln1852_Arg1858del). The variants were reported *de novo*, with paternity having been confirmed, suggesting the occurrence of germline mosaicism in MKHK-ID4.

The MKHK-ID4 episignature was found in all 21 tested individuals with a variant in the ID4 domain, but, surprisingly, also in C.ZT.1 and (although moderately) in C.ZT.2, who both had a variant in between the ZZ and TAZ2 domains. Consistent with these finding, they both showed features comparable to the MKHK-ID4 group (Figure 1). We hypothesize that variants in this specific area may affect the same functions as variants within ID4. AlphaFold models did not show proximity of these variants to the ID4 helix (Figure 3). Further studies are needed to elucidate the underlying mechanisms involved.

We previously claimed that the borders of the MKHK region were located at the N-terminal region of CBP (GenBank: NM_004380.2 and NP_004371.2) between bp 5,094 and 5,128 (residues 1,698–1,710) and at the C-terminal region between bp 5,614 and 5,641 (residues 1,872–1,881).[6] Based on our current findings, we suggest that the borders are closely consistent with the region that spans the ZZ and TAZ2 domains and the first α helix of ID4 (residues 1,705–1,875 for CBP, NP_004371.2, and 1,668–1,833 for p300, NP_001420.2) which is supported by the episignature results in our study showing MKHK methylation profiles for individuals with variants between residue 1,710 and 1,872 in CBP. However, we found three different methylation profiles within this area and one could argue that in fact (at least) three separate syndromes are found, rather than three subtypes of MKHK. As many individuals already received the diagnosis of MKHK, and Menke-Hennekam syndrome has previously been identified as an umbrella term for various disorders,[7] it may be more accurate to identify the individuals as having MKHK-ZZ-, MKHK-TAZ2-, and MKHK-ID4-related

syndromes. We cannot, however, rule out that other subcategories will be needed, in case future individuals show that there are separate entities, even within these domains/regions. The individuals with variants between ZZ and TAZ2 and phenotypes and episignatures fitting MKHK-ID4 demonstrate the complex functions and 3D structure of CBP/p300. We also cannot exclude that patients with MKHK methylation profiles and phenotypes will be found outside the borders of MKHK in the future when more individuals are studied. Nishi et al. reported on three individuals with frameshift variants in or near the nuclear receptor coactivator (NR) region at the C-terminal end of *CREBBP*.[41] As they did not show all the typical features of RSTS, the authors suggested the diagnosis of MKHK and that MKHK could possibly also be caused by variants beyond the first α helix of ID4. However, we think that the individuals had RSTS, based on their phenotypes and genotypes, with frameshift variants typically resulting in loss of function and thus RSTS. Additionally, variants in and near the NR region of *CREBBP* had previously been found to cause RSTS.[6] In a case report from 2021, Wang et al.[13] reported on a young girl with a variant in the HAT domain of *CREBBP* (p.(Phe1633del)) whose features partially overlapped those of MKHK-ID4. We recently reviewed data of a girl with a similar phenotype and the same missense mutation, with neither an RSTS episignature nor a clear MKHK episignature or methylation profile. However, increased numbers are needed to further elucidate whether this, and maybe variants elsewhere, may give rise to (additional subtypes of) MKHK. In ambiguous cases such as the ones described above, episignatures for RSTS and MKHK-ID4[11] will offer a valuable tool in differentiating between MKHK-ID4 and RSTS syndromes. Also, in case of VUSs within the MKHK-ID4 region, episignatures offer a functional assay to help reclassify ambiguous genetic findings.[11] While we present evidence of a milder methylation profile for the ZZ and TAZ2 domains, further work, including the expansion of each cohort, is required to refine the classifier to meet the performance and reproducibility of an episignature biomarker.

This study demonstrates that MKHK consists of at least three subtypes (MKHK-ZZ, MKHK-TAZ2, and MKHK-ID4), based on distinct phenotypes and domain-specific methylation profiles. DNA methylation episignatures enable stratification of molecular pathophysiologic entities within a gene or across a family of paralogous genes.

Data and code availability

Some of the datasets used in this study are available publicly as previously described (Aref-Eshghi et al.[32]). The raw DNA methylation data for other samples are not available due to institutional and ethics restrictions. The software used in this study is publicly available with software packages and versions described in the section "subjects, material, and methods."

Consortia

The members of the MKHK Research Consortium are Andrea Angius, Janice A. Baker, Emma Bedoukian, Vikas Bhambhani, Olaf Bodamer, Alan O'Brien, Jill Clayton-Smith, Laura Crisponi, Anna María Cueto González, the DDD study, Koenraad Devriendt, Elena Dominguez Garrido; Nadja Ehmke, Albertien van Eerde, Annette P.M. van den Elzen, Laurence Faivre, Laura Fisher, Josue A. Flores-Daboub, Alison Foster, Jennifer Friedman, Elisabeth Gabau, Elena Galazzi, Sixto García-Miñaúr, Livia Garavelli, Thatjana Gardeitchik, Erica H. Gerkes, Julien van Gils, Jacques C. Giltay, Aixa Gonzalez Garcia, Ketil Riddervold Heimdal, Denise Horn, Gunnar Houge, Sophia B. Hufnagel, Denisa Ilencikova, Sophie Julia, Sarina G. Kant, Esther Kinning, Eric W. Klee, Chelsea Kois, Maja Kovačević, A.M.A. (Guus) Lachmeijer, Brendan Lanpher, Marine Lebrun, Eyby Leon, Angie Ward Lichty, Ruth Lin, Isabel Llano-Rivas, Sally Ann Lynch, Saskia M. Maas, Silvia B. Maitz, Shane McKee, Daniela Melis, Elisabetta Merati, Giuseppe Merla, Ruth Newbury-Ecob, Mathilde Nizon, Soo-Mi Park, Jennifer Patterson, Florence Petit, Hilde Peeters, Luca Persani, Ivana Persico, Valentina Pes, Marzia Pollazzon, Thomas Potjer, Lorraine Potocki, Carrie Pottinger, Chitra Prasad, Eloise J. Prijoles, Nicola K. Ragge, Jan Peter Rake, Conny M.A. van Ravenswaaij-Arts, Gillian Rea, Claudia Ruivenkamp, Audrey Rutz, Sulagna C Saitta, Rossana Sanchez Russo, Gijs W.E. Santen, Elise Schaefer, Vandana Shashi, Laura Schultz-Rogers, Andrea Sluga, Stefano Sotgiu, Elisabeth Steichen-Gersdorf, Jennifer A. Sullivan, Yu Sun, Mohnish Suri, Marco Tartaglia, Matt Tedder, Paulien Terhal, Ian Tully, Nienke Verbeek, Maren Wenzel, Susan M. White, Bing Xiao.

Web resources

AlphaFill: https://alphafill.eu.

AlphaFold: https://alphafold.ebi.ac.uk.

ClinVar-NCBI: https://wwwncbi.nlm.nih.gov/clinvar/

DECIPHER v1113: https://deciphergenomics.org.

EpiSign Knowledge Database: https://episign.lhsc.on.ca/index.html.

GnomAD: https://gnomad.broadinstitute.org.

NCBI CBP: https://wwwncbinlmnihgov/protein/NP_0043712.

NCBI p300: https://wwwncbinlmnihgov/protein/NP_0014202.

OMIM, OMIM: http://www.omim.org/

UniProt: https://www.uniprot.org/uniprotkb.

Supplemental information

Supplemental information can be found online at https://doi.org/10.1016/j.xhgg.2024.100287.

Acknowledgments

We are pleased to thank all families for their generous participation. We thank Raoul Hennekam for useful discussions. We thank

René Minnaar and Hester Heidinga, Amsterdam UMC, for their support in sample handling. L.A.M. obtained funding from Asociación las mil batallas de Claudia; Kortabitarte Bilbao, familia eta lagunak; Emma Children's hospital foundation (WAR2018-05), and Janivo Foundation (2018241). B.S. received funding from the government of Canada through Genome Canada and the Ontario Genomics Institute (OGI-188). This work was partially supported by funds from the Italian Ministry of Health, Rome, Italy (02C502_2005 to L.P.). S.B. was supported by the NIHR Manchester Biomedical Research Centre (NIHR203308). This work was generated within the European Reference Network ITHACA.

Author contributions

Conceptualization, S.H., H.J.B., J.M.S., S.P.R., B.S., and L.A.M.; methodology, S.H., H.J.B., J.M.S., H.M., Z.A.J., B.H., E.M.W., M.A., S.P.R., B.S., and L.A.M.; investigation, S.H., H.J.B., J.M.S., M.A.L., J.K., B.S., and L.A.M.; formal analysis, S.H., H.J.B., J.M.S., Z.A.J., B.J.H., E.M.W., and L.A.M.; methylation analysis, S.H., H.M.C., M.A., M.A.L., and B.S.; patient recruitment, clinical and diagnostic evaluations, H.J.B., J.M.S., L.A.M., and all other co-authors contributed by sharing clinical information; data curation, S.H., H.J.B., J.M.S., L.A.M., and J.K.; writing – original draft, S.H., H.J.B., J.M.S., and L.A.M.; writing – review & editing, all authors; supervision, S.P.R., V.d.W., B.S., and L.A.M.; funding acquisition, B.S. and L.A.M.

Declaration of interests

B.S. is a shareholder in EpiSign Inc., a biotech firm involved in commercial application of EpiSign technology.

Received: October 20, 2023
Accepted: March 26, 2024

References

1. Petrij, F., Giles, R.H., Dauwerse, H.G., Saris, J.J., Hennekam, R.C., Masuno, M., Tommerup, N., van Ommen, G.J., Goodman, R.H., Peters, D.J., et al. (1995). Rubinstein-Taybi syndrome caused by mutations in the transcriptional co-activator CBP. Nature *376*, 348–351. https://doi.org/10.1038/376348a0.
2. Roelfsema, J.H., White, S.J., Ariyürek, Y., Bartholdi, D., Niedrist, D., Papadia, F., Bacino, C.A., den Dunnen, J.T., van Ommen, G.J.B., Breuning, M.H., et al. (2005). Genetic heterogeneity in Rubinstein-Taybi syndrome: mutations in both the CBP and EP300 genes cause disease. Am. J. Hum. Genet. *76*, 572–580. https://doi.org/10.1086/429130.
3. Cross, E., Duncan-Flavell, P.J., Howarth, R.J., Hobbs, J.I., Thomas, N.S., and Bunyan, D.J. (2020). Screening of a large Rubinstein-Taybi cohort identified many novel variants and emphasizes the importance of the CREBBP histone acetyltransferase domain. Am. J. Med. Genet. *182*, 2508–2520. https://doi.org/10.1002/ajmg.a.61813.
4. Hennekam, R.C.M. (2006). Rubinstein-Taybi syndrome. Eur. J. Hum. Genet. *14*, 981–985. https://doi.org/10.1038/sj.ejhg.5201594.
5. Menke, L.A., van Belzen, M.J., Alders, M., Cristofoli, F., DDD Study, Ehmke, N., Fergelot, P., Foster, A., Gerkes, E.H., Hoffer, M.J.V., et al. (2016). CREBBP mutations in individuals without Rubinstein-Taybi syndrome phenotype. Am. J. Med. Genet. *170*, 2681–2693. https://doi.org/10.1002/ajmg.a.37800.
6. Menke, L.A., DDD study, Gardeitchik, T., Hammond, P., Heimdal, K.R., Houge, G., Hufnagel, S.B., Ji, J., Johansson, S., Kant, S.G., et al. (2018). Further delineation of an entity caused by CREBBP and EP300 mutations but not resembling Rubinstein-Taybi syndrome. Am. J. Med. Genet. *176*, 862–876. https://doi.org/10.1002/ajmg.a.38626.
7. Banka, S., Sayer, R., Breen, C., Barton, S., Pavaine, J., Sheppard, S.E., Bedoukian, E., Skraban, C., Cuddapah, V.A., and Clayton-Smith, J. (2019). Genotype-phenotype specificity in Menke-Hennekam syndrome caused by missense variants in exon 30 or 31 of CREBBP. Am. J. Med. Genet. *179*, 1058–1062. https://doi.org/10.1002/ajmg.a.61131.
8. Ponting, C.P., Blake, D.J., Davies, K.E., Kendrick-Jones, J., and Winder, S.J. (1996). ZZ and TAZ: new putative zinc fingers in dystrophin and other proteins. Trends Biochem. Sci. *21*, 11–13.
9. De Guzman, R.N., Liu, H.Y., Martinez-Yamout, M., Dyson, H.J., and Wright, P.E. (2000). Solution structure of the TAZ2 (CH3) domain of the transcriptional adaptor protein CBP. J. Mol. Biol. *303*, 243–253. https://doi.org/10.1006/jmbi.2000.4141.
10. Piai, A., Calçada, E.O., Tarenzi, T., Grande, A.D., Varadi, M., Tompa, P., Felli, I.C., and Pierattelli, R. (2016). Just a Flexible Linker? The Structural and Dynamic Properties of CBP-ID4 Revealed by NMR Spectroscopy. Biophys. J. *110*, 372–381. https://doi.org/10.1016/j.bpj.2015.11.3516.
11. Levy, M.A., McConkey, H., Kerkhof, J., Barat-Houari, M., Bargiacchi, S., Biamino, E., Bralo, M.P., Cappuccio, G., Ciolfi, A., Clarke, A., et al. (2022). Novel diagnostic DNA methylation episignatures expand and refine the epigenetic landscapes of Mendelian disorders. HGG Adv. *3*, 100075. https://doi.org/10.1016/j.xhgg.2021.100075.
12. Angius, A., Uva, P., Oppo, M., Persico, I., Onano, S., Olla, S., Pes, V., Perria, C., Cuccuru, G., Atzeni, R., et al. (2019). Confirmation of a new phenotype in an individual with a variant in the last part of exon 30 of CREBBP. Am. J. Med. Genet. *179*, 634–638. https://doi.org/10.1002/ajmg.a.61052.
13. Wang, Q., Xu, W., Liu, Y., and Yuan, H. (2021). A Novel CREBBP in-Frame Deletion Variant in a Chinese Girl with Atypical Rubinstein-Taybi Syndrome Phenotypes. J. Mol. Neurosci. *71*, 607–612. https://doi.org/10.1007/s12031-020-01681-x.
14. Sima, A., Smădeanu, R.E., Simionescu, A.A., Nedelea, F., Vlad, A.M., and Becheanu, C. (2022). Menke-Hennekam Syndrome: A Literature Review and a New Case Report. Children *9*, 759. https://doi.org/10.3390/children9050759.
15. Landrum, M.J., Lee, J.M., Benson, M., Brown, G.R., Chao, C., Chitipiralla, S., Gu, B., Hart, J., Hoffman, D., Jang, W., et al. (2018). ClinVar: improving access to variant interpretations and supporting evidence. Nucleic Acids Res. *46*, D1062–D1067. https://doi.org/10.1093/nar/gkx1153.
16. Firth, H.V., Richards, S.M., Bevan, A.P., Clayton, S., Corpas, M., Rajan, D., Van Vooren, S., Moreau, Y., Pettett, R.M., and Carter, N.P. (2009). DECIPHER: Database of Chromosomal Imbalance and Phenotype in Humans Using Ensembl Resources. Am. J. Hum. Genet. *84*, 524–533. https://doi.org/10.1016/j.ajhg.2009.03.010.
17. Karczewski, K.J., Francioli, L.C., Tiao, G., Cummings, B.B., Alföldi, J., Wang, Q., Collins, R.L., Laricchia, K.M., Ganna, A., Birnbaum, D.P., et al. (2019). Variation across 141,456 human exomes and genomes reveals the spectrum of loss-of-function intolerance across human protein-coding genes. Preprint at bioRxiv *5*. https://doi.org/10.1101/531210.

18. Pollard, K.S., Hubisz, M.J., Rosenbloom, K.R., and Siepel, A. (2010). Detection of nonneutral substitution rates on mammalian phylogenies. Genome Res. *20*, 110–121. https://doi.org/10.1101/gr.097857.109.
19. Danecek, P., Bonfield, J.K., Liddle, J., Marshall, J., Ohan, V., Pollard, M.O., Whitwham, A., Keane, T., McCarthy, S.A., Davies, R.M., and Li, H. (2021). Twelve years of SAMtools and BCFtools. GigaScience *10*, giab008. https://doi.org/10.1093/gigascience/giab008.
20. Ng, P.C., and Henikoff, S. (2003). SIFT: Predicting amino acid changes that affect protein function. Nucleic Acids Res. *31*, 3812–3814. https://doi.org/10.1093/nar/gkg509.
21. Adzhubei, I.A., Schmidt, S., Peshkin, L., Ramensky, V.E., Gerasimova, A., Bork, P., Kondrashov, A.S., and Sunyaev, S.R. (2010). A method and server for predicting damaging missense mutations. Nat. Methods *7*, 248–249. https://doi.org/10.1038/nmeth0410-248.
22. Rentzsch, P., Witten, D., Cooper, G.M., Shendure, J., and Kircher, M. (2019). CADD: predicting the deleteriousness of variants throughout the human genome. Nucleic Acids Res. *47*. D886-d894. https://doi.org/10.1093/nar/gky1016.
23. Katsonis, P., Wilhelm, K., Williams, A., and Lichtarge, O. (2022). Genome interpretation using in silico predictors of variant impact. Hum. Genet. *141*, 1549–1577. https://doi.org/10.1007/s00439-022-02457-6.
24. Ioannidis, N.M., Rothstein, J.H., Pejaver, V., Middha, S., McDonnell, S.K., Baheti, S., Musolf, A., Li, Q., Holzinger, E., Karyadi, D., et al. (2016). REVEL: An Ensemble Method for Predicting the Pathogenicity of Rare Missense Variants. Am. J. Hum. Genet. *99*, 877–885. https://doi.org/10.1016/j.ajhg.2016.08.016.
25. Samocha, K.E., Kosmicki, J.A., Karczewski, K.J., O'Donnell-Luria, A.H., Pierce-Hoffman, E., MacArthur, D.G., Neale, B.M., and Daly, M.J. (2017). Regional missense constraint improves variant deleteriousness prediction. Preprint at bioRxiv *1*. https://doi.org/10.1101/148353.
26. Gunning, A.C., Fryer, V., Fasham, J., Crosby, A.H., Ellard, S., Baple, E.L., and Wright, C.F. (2021). Assessing performance of pathogenicity predictors using clinically relevant variant datasets. J. Med. Genet. *58*, 547–555. https://doi.org/10.1136/jmedgenet-2020-107003.
27. Tian, Y., Pesaran, T., Chamberlin, A., Fenwick, R.B., Li, S., Gau, C.L., Chao, E.C., Lu, H.M., Black, M.H., and Qian, D. (2019). REVEL and BayesDel outperform other in silico meta-predictors for clinical variant classification. Sci. Rep. *9*, 12752. https://doi.org/10.1038/s41598-019-49224-8.
28. Richards, S., Aziz, N., Bale, S., Bick, D., Das, S., Gastier-Foster, J., Grody, W.W., Hegde, M., Lyon, E., Spector, E., et al. (2015). Standards and guidelines for the interpretation of sequence variants: a joint consensus recommendation of the American College of Medical Genetics and Genomics and the Association for Molecular Pathology. Genet. Med. *17*, 405–424. https://doi.org/10.1038/gim.2015.30.
29. Aref-Eshghi, E., Bend, E.G., Hood, R.L., Schenkel, L.C., Carere, D.A., Chakrabarti, R., Nagamani, S.C.S., Cheung, S.W., Campeau, P.M., Prasad, C., et al. (2018). BAFopathies' DNA methylation episignatures demonstrate diagnostic utility and functional continuum of Coffin-Siris and Nicolaides-Baraitser syndromes. Nat. Commun. *9*, 4885. https://doi.org/10.1038/s41467-018-07193-y.
30. Aryee, M.J., Jaffe, A.E., Corrada-Bravo, H., Ladd-Acosta, C., Feinberg, A.P., Hansen, K.D., and Irizarry, R.A. (2014). Minfi: a flexible and comprehensive Bioconductor package for the analysis of Infinium DNA methylation microarrays. Bioinformatics *30*, 1363–1369. https://doi.org/10.1093/bioinformatics/btu049.
31. Houseman, E.A., Accomando, W.P., Koestler, D.C., Christensen, B.C., Marsit, C.J., Nelson, H.H., Wiencke, J.K., and Kelsey, K.T. (2012). DNA methylation arrays as surrogate measures of cell mixture distribution. BMC Bioinf. *13*, 86. https://doi.org/10.1186/1471-2105-13-86.
32. Aref-Eshghi, E., Kerkhof, J., Pedro, V.P., Groupe DI France, Barat-Houari, M., Ruiz-Pallares, N., Andrau, J.C., Lacombe, D., Van-Gils, J., Fergelot, P., et al. (2020). Evaluation of DNA Methylation Episignatures for Diagnosis and Phenotype Correlations in 42 Mendelian Neurodevelopmental Disorders. Am. J. Hum. Genet. *106*, 356–370. https://doi.org/10.1016/j.ajhg.2020.01.019.
33. Aref-Eshghi, E., Bend, E.G., Colaiacovo, S., Caudle, M., Chakrabarti, R., Napier, M., Brick, L., Brady, L., Carere, D.A., Levy, M.A., et al. (2019). Diagnostic Utility of Genome-wide DNA Methylation Testing in Genetically Unsolved Individuals with Suspected Hereditary Conditions. Am. J. Hum. Genet. *104*, 685–700. https://doi.org/10.1016/j.ajhg.2019.03.008.
34. Peters, T.J., Buckley, M.J., Statham, A.L., Pidsley, R., Samaras, K., V Lord, R., Clark, S.J., and Molloy, P.L. (2015). De novo identification of differentially methylated regions in the human genome. Epigenet. Chromatin *8*, 6. https://doi.org/10.1186/1756-8935-8-6.
35. Dyson, H.J., and Wright, P.E. (2016). Role of Intrinsic Protein Disorder in the Function and Interactions of the Transcriptional Coactivators CREB-binding Protein (CBP) and p300. J. Biol. Chem. *291*, 6714–6722. https://doi.org/10.1074/jbc.R115.692020.
36. Ramos, Y.F.M., Hestand, M.S., Verlaan, M., Krabbendam, E., Ariyurek, Y., van Galen, M., van Dam, H., van Ommen, G.J.B., den Dunnen, J.T., Zantema, A., and 't Hoen, P.A.C. (2010). Genome-wide assessment of differential roles for p300 and CBP in transcription regulation. Nucleic Acids Res. *38*, 5396–5408. https://doi.org/10.1093/nar/gkq184.
37. Sheahan, T.W., Major, V., Webb, K.M., Bryan, E., and Voigt, P. (2020). The TAZ2 domain of CBP/p300 directs acetylation towards H3K27 within chromatin. Preprint at bioRxiv *1*. https://doi.org/10.1101/2020.07.21.214338.
38. Ibrahim, Z., Wang, T., Destaing, O., Salvi, N., Hoghoughi, N., Chabert, C., Rusu, A., Gao, J., Feletto, L., Reynoird, N., et al. (2022). Structural insights into p300 regulation and acetylation-dependent genome organisation. Preprint at bioRxiv *2*. https://doi.org/10.1101/2022.03.14.484228.
39. Levy, M.A., Relator, R., McConkey, H., Pranckeviciene, E., Kerkhof, J., Barat-Houari, M., Bargiacchi, S., Biamino, E., Palomares Bralo, M., Cappuccio, G., et al. (2022). Functional correlation of genome-wide DNA methylation profiles in genetic neurodevelopmental disorders. Hum. Mutat. *43*, 1609–1628. https://doi.org/10.1002/humu.24446.
40. Yampolsky, L.Y., and Stoltzfus, A. (2005). The exchangeability of amino acids in proteins. Genetics *170*, 1459–1472. https://doi.org/10.1534/genetics.104.039107.
41. Nishi, E., Takenouchi, T., Miya, F., Uehara, T., Yanagi, K., Hasegawa, Y., Ueda, K., Mizuno, S., Kaname, T., Kosaki, K., and Okamoto, N. (2022). The novel and recurrent variants in exon 31 of CREBBP in Japanese patients with Menke-Hennekam syndrome. Am. J. Med. Genet. *188*, 446–453. https://doi.org/10.1002/ajmg.a.62533.

HGGA, Volume *5*

Supplemental information

Menke-Hennekam syndrome; delineation of domain-specific subtypes with distinct clinical and DNA methylation profiles

Sadegheh Haghshenas, Hidde J. Bout, Josephine M. Schijns, Michael A. Levy, Jennifer Kerkhof, Pratibha Bhai, Haley McConkey, Zandra A. Jenkins, Ella M. Williams, Benjamin J. Halliday, Sylvia A. Huisman, Peter Lauffer, Vivian de Waard, Laura Witteveen, Siddharth Banka, Angela F. Brady, Elena Galazzi, Julien van Gils, Anna C.E. Hurst, Frank J. Kaiser, Didier Lacombe, Antonio F. Martinez-Monseny, Patricia Fergelot, Fabíola P. Monteiro, Ilaria Parenti, Luca Persani, Fernando Santos-Simarro, Brittany N. Simpson, MKHK Research Consortium, Mariëlle Alders, Stephen P. Robertson, Bekim Sadikovic, and Leonie A. Menke

Table of contents

Figure S1. Lateral facial and distal limb morphology of the presently described individuals with a variant in the MKHK region of *CREBBP* and *EP300*. 2

Table S1. Summary of the 3-step process for the selection of probes 8

Table S2. Morphological characteristics of individuals with a confirmed MKHK methylation profile or episignature (in ID4) per MKHK subtype. 8

Table S3 (see excel file). Clinical characteristics and morphological features of the presently described individuals with a variant in the MKHK region of *CREBBP* and *EP300*. 9

Table S4 (see excel file). Genotypes, episignatures and in silico prediction scores of all presently described individuals with a variant in the MKHK region of CREBBP (NM_004380.2) or EP300 (NM_001429.3). 9

Table S5. List of identified differentially methylated regions. 10

References 12

Figure S1. Lateral facial and distal limb morphology of the presently described individuals with a variant in the MKHK region of *CREBBP* and *EP300*.

The lateral facial characteristics, the hands and feet of individuals with a variant in (A) the ZZ-domain, (B) TAZ2-domain, (C) ID4, and (D) in between the ZZ and TAZ2-domain (C.ZT.1 en C.ZT.2) and in between TAZ2 and ID4 (C.TI.29). Study numbers are depicted within each photograph reflecting the affected gene (C or E representing *CREBBP* and *EP300*) and domain (Z, T, I, ZT, and TI representing ZZ, TAZ2, ID4, the region between ZZ and TAZ2, and the region between TAZ2 and ID4, respectively) followed by a unique number. The study numbers of individuals with a confirmed MKHK episignature or methylation profile are displayed in bold, and of individuals who were tested, but in whom no MKHK episignature or methylation profile was found, in italics. Detailed description of facial and distal limb morphology of all individuals can be found in Table S3.

A

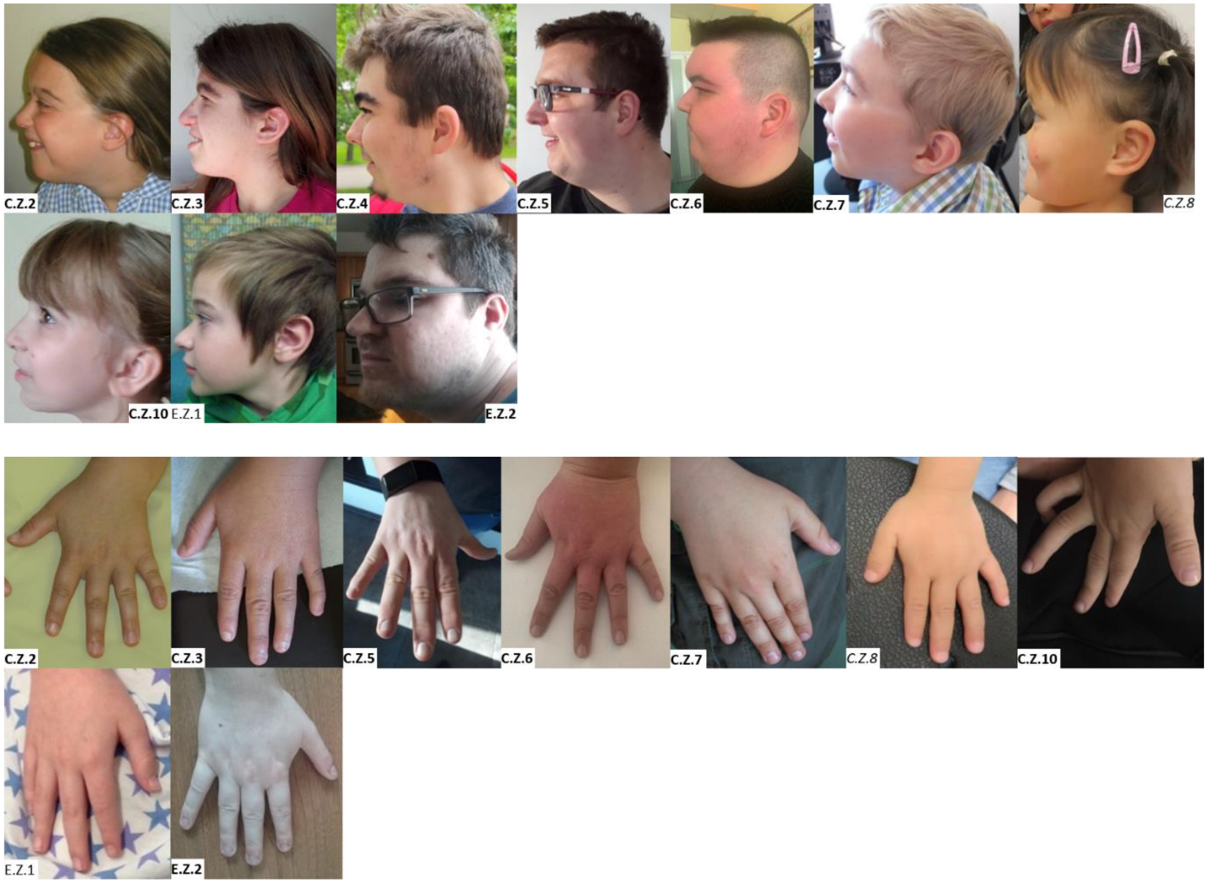

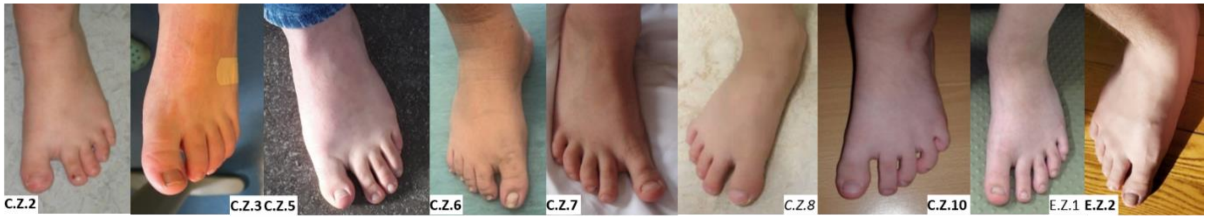
C.Z.2
C.Z.3
C.Z.5
C.Z.6
C.Z.7
C.Z.8
C.Z.10
E.Z.1
E.Z.2

B

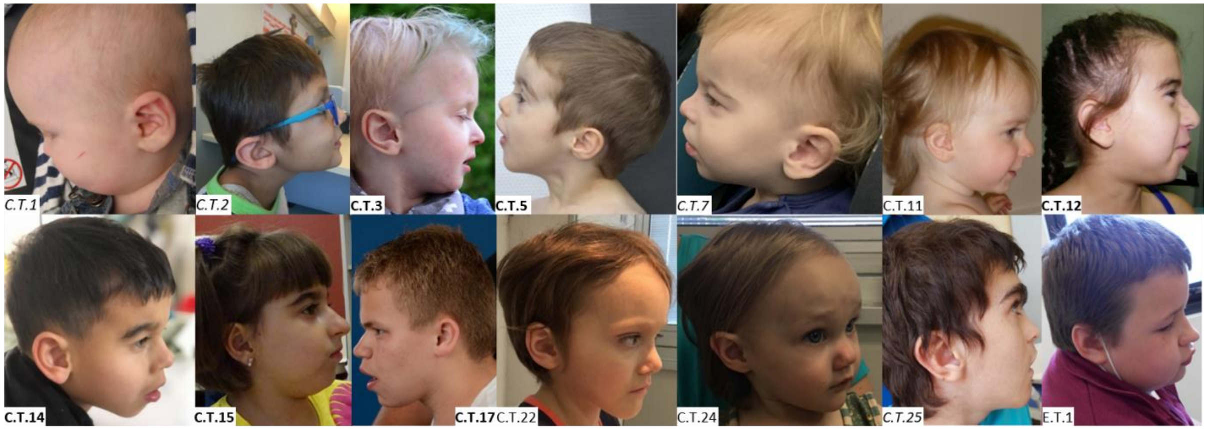
C.T.1
C.T.2
C.T.3
C.T.5
C.T.7
C.T.11
C.T.12
C.T.14
C.T.15
C.T.17
C.T.22
C.T.24
C.T.25
E.T.1

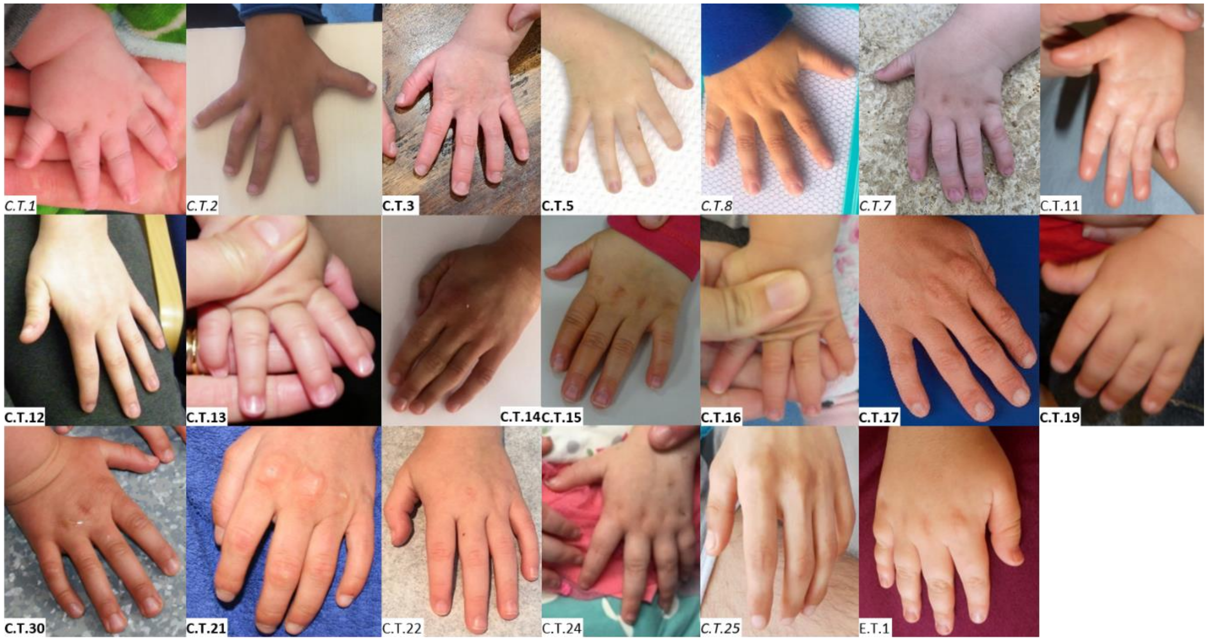
C.T.1
C.T.2
C.T.3
C.T.5
C.T.8
C.T.7
C.T.11
C.T.12
C.T.13
C.T.14
C.T.15
C.T.16
C.T.17
C.T.19
C.T.30
C.T.21
C.T.22
C.T.24
C.T.25
E.T.1

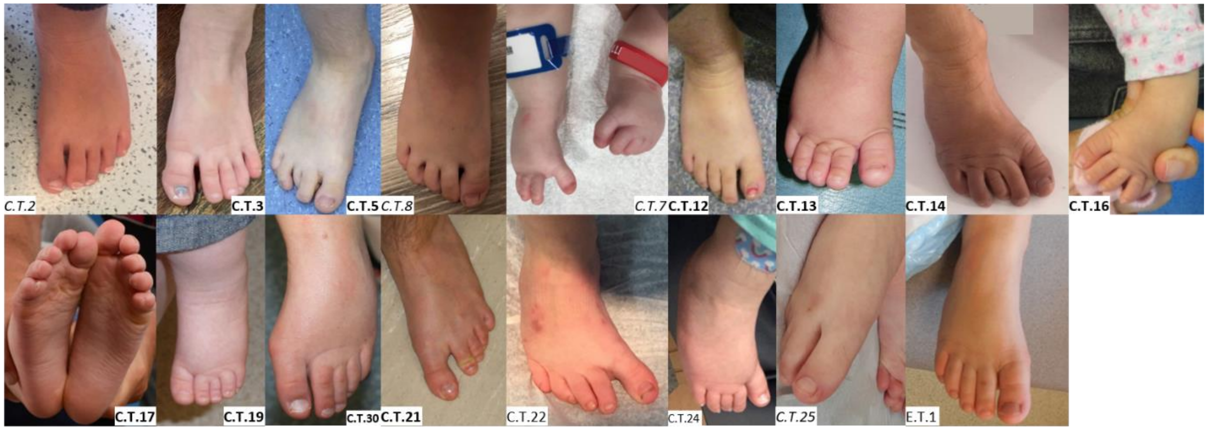
C.T.2
C.T.3
C.T.5
C.T.8
C.T.7
C.T.12
C.T.13
C.T.14
C.T.16
C.T.17
C.T.19
C.T.30
C.T.21
C.T.22
C.T.24
C.T.25
E.T.1

C

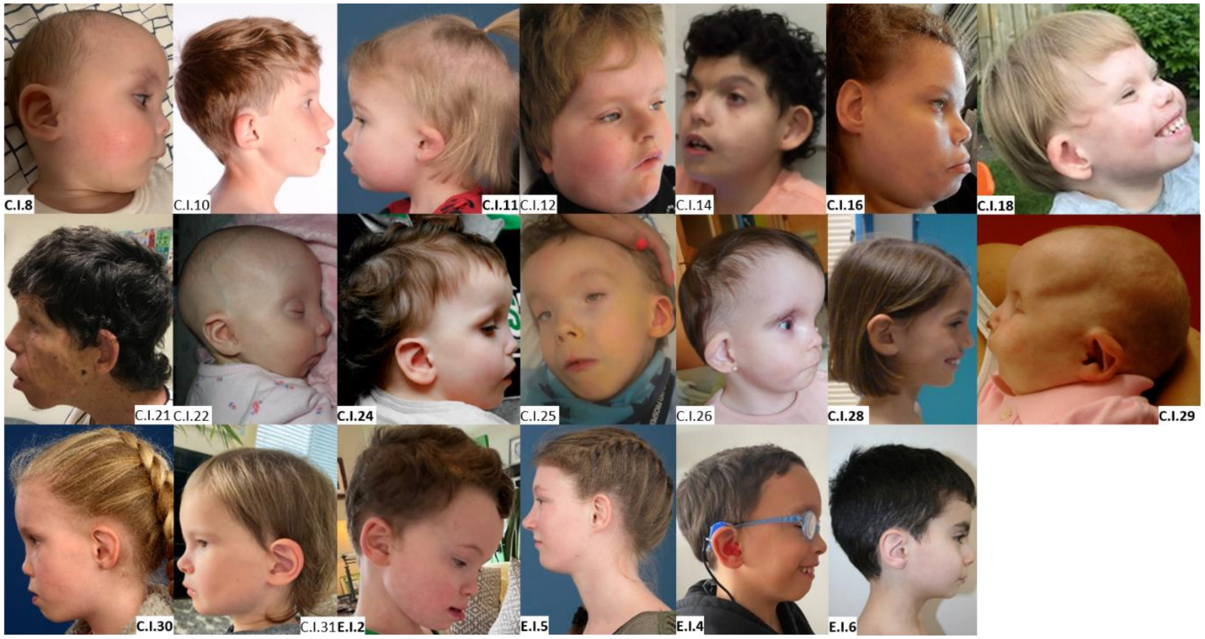
C.I.8
C.I.10
C.I.11
C.I.12
C.I.14
C.I.16
C.I.18
C.I.21
C.I.22
C.I.24
C.I.25
C.I.26
C.I.28
C.I.29
C.I.30
C.I.31
E.I.2
E.I.5
E.I.4
E.I.6

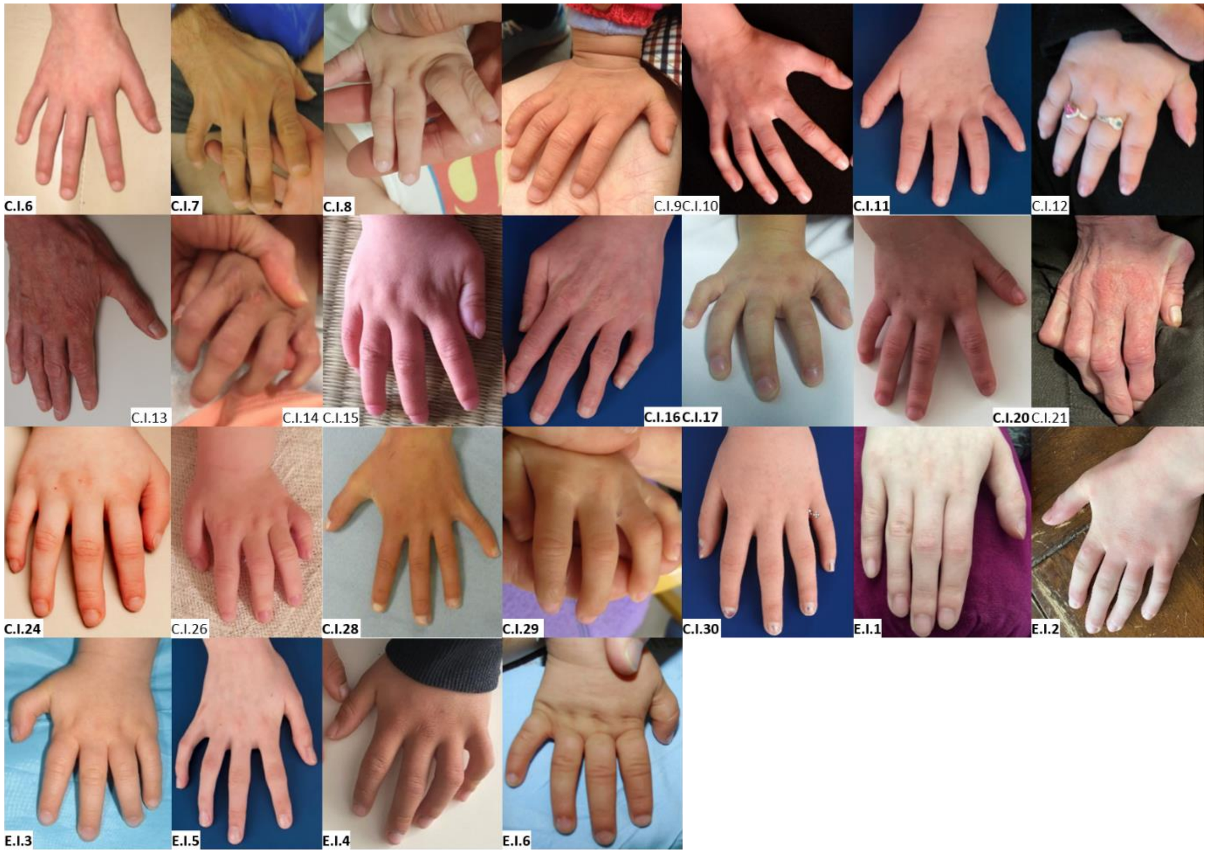
C.I.6
C.I.7
C.I.8
C.I.9
C.I.10
C.I.11
C.I.12
C.I.13
C.I.14
C.I.15
C.I.16
C.I.17
C.I.20
C.I.21
C.I.24
C.I.26
C.I.28
C.I.29
C.I.30
E.I.1
E.I.2
E.I.3
E.I.5
E.I.4
E.I.6

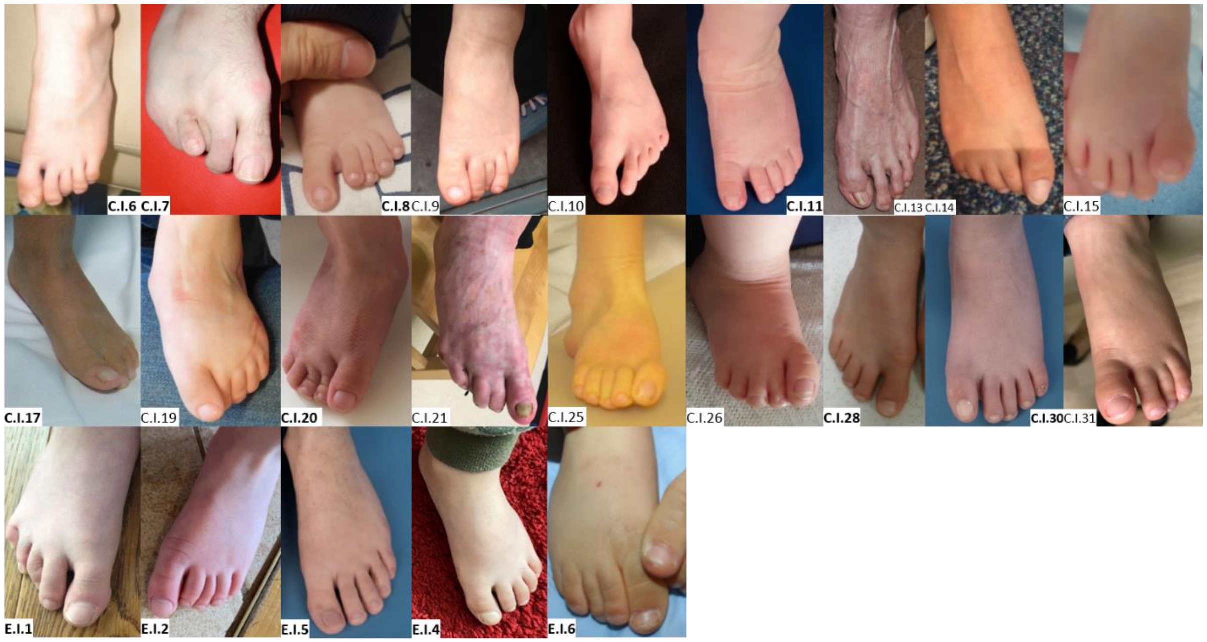
C.I.6
C.I.7
C.I.8
C.I.9
C.I.10
C.I.11
C.I.13
C.I.14
C.I.15
C.I.17
C.I.19
C.I.20
C.I.21
C.I.25
C.I.26
C.I.28
C.I.30
C.I.31
E.I.1
E.I.2
E.I.5
E.I.4
E.I.6

D

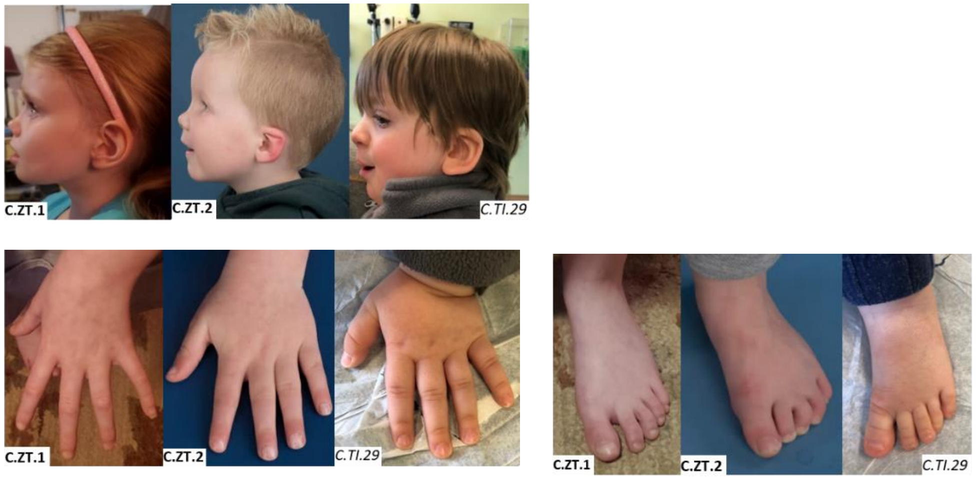
C.ZT.1
C.ZT.2
C.TI.29
C.ZT.1
C.ZT.2
C.TI.29
C.ZT.1
C.ZT.2
C.TI.29

Table S1. Summary of the 3-step process for the selection of probes

Episignature	Number of probes after the first step	Number of probes after the second step	Cut-off value for correlation
MKHK_ZZ	1000	333	0.85
MKHK_TAZ2	900	225	0.75
MKHK_ID4	900	225	0.75

Table S2. Morphological characteristics of individuals with a confirmed MKHK methylation profile or episignature (in ID4) per MKHK subtype.

ZZ, zinc finger ZZ-type; TAZ2, zinc finger TAZ-type; ID4, first α-helix of the fourth intrinsically disordered region of CBP/p300.

	**MKHK-ZZ (n=9)**	**MKHK-TAZ2 (n=14)**	**MKHK-ID4 (n=21)**
**Sparse hair**	0/9	3/12 (25%)	10/19 (53%)
**Prominent forehead**	2/9 (22%)	4/13 (31%)	15/20(75%)
**Face, square (FS) or flat (FF)**	FF 1/9 (11%)<br>FS 1/9 (11%)	FF 1/13 (8%)<br>FS 2/13 (15%)	FF 6/20 (30%)<br>FS 6/20 (30%)
**Thick eyebrows**	5/9 (56%)	7/13 (54%)	6/21 (29%)
**Flared eyebrows**	5/9 (56%)	0/13	0/18
**Telecanthi (T)/ epicanthi (E)**	T 2/9 (22%)<br>E 2/9 (22%)	T 2/14 (14%)<br>E 5/14 (36%)	T 7/21 (33%)<br>E 7/21 (33%)
**Palpebral fissures Up (U)- or downslanted (D)**	U 0/7<br>D 1/7 (14%)	U 1/13 (8%)<br>D 2/13 (15%)	U 15/21 (71%)<br>D 0/21
**Short palpebral fissures**	1/9 (11%)	2/14 (14%)	15/21 (71%)
**Ptosis/ Blepharophimosis**	4/8 (50%)	5/11 (45%)	15/21 (71%)
**Long eyelashes**	3/9 (33%)	5/12 (42%)	5/20 (25%)
**Ears Low-set (L) / Short (S)**	L 0/8<br>S 0/8	L 2/9 (22%)<br>S 1/9 (11%)	L 6/19 (32%)<br>S 1/19 (5%)
**Protruding ears (upper part)**	0/9	3/12 (25%)	13/20 (65%)
**Overfolded helix**	1/8 (13%)	1/11 (9%)	8/19 (42%)
**Absent earlobe**	0/8	1/11 (9%)	2/19 (10%)
**Full cheeks**	4/9 (44%)	3/14 (21%)	10/21 (48%)
**Malar flattening**	0/9	1/12 (8%)	8/18 (44%)
**Short nose**	2/9 (22%)	2/14 (14%)	11/21 (52%)
**Depressed nasal bridge**	1/9 (11%)	0/12	9/18 (50%)
**Narrow nasal bridge**	1/9 (11%)	2/14 (14%)	2/21 (9%)
**Depressed nasal ridge**	0/9	1/12 (7%)	10/18 (56%)
**Broad nasal tip**	3/9 (33%)	8/14 (57%)	12/21 (57%)
**Short columella**	0/8	3/13 (23%)	14/21 (67%)
**Anteverted nares**	1/9 (11%)	4/14 (29%)	11/21 (52%)
**Underdeveloped alae nasi**	0/9	1/13 (8%)	1/21 (5%)
**Philtrum Short (S)/Long (L)/Deep (D)**	S 1/9 (11%)<br>L 1/9 (11%)<br>D 0	S 0/13<br>L 5/13 (38%)<br>D 1/13 (8%)	S 1/21 (5%)<br>L 13/21 (62%)<br>D 4/21 (19%)
**Everted vermilion of upper lip**	1/9 (11%)	2/13 (15%)	4/21 (19%)
**Thin vermilion of upper lip**	5/9 (56%)	4/13 (31%)	6/21 (29%)
**High palate**	6/7 (86%)	1/10 (10%)	10/17 (59%)
**Missing teeth**	2/8 (25%)	0/8	3/10 (30%)
**Retro- or micrognathia**	1/9 (11%)	5/12 (42%)	5/19 (26%)
**Ulnar deviation of finger(s)**	0/7	0/13	3/20 (15%)
**Clinodactyly fifth finger**	1/9 (11%)	2/11 (18%)	8/20 (40%)
**Tapering fingers**	0/6	0/13	2/20 (10%)
**Prominent digital pads**	0/6	1/8 (13%)	4/15 (27%)
**Sandal gap**	3/6 (50%)	3/11 (27%)	5/18 (28%)
**Overlapping toes**	0/6	3/13 (23%)	6/19 (32%)
**Halluces Broad (B)/Narrow (N)**	0/5	B 2/12 (17%)<br>N 1/12 (8%)	B 3/18 (17%)<br>N 1/18 (6%)
**Fibular deviation distal phalanx halluces**	0/6	2/12 (17%)	8/20 (40%)
**Cutaneous partial syndactyly of toes**	0/6	2/12 (17%)	5/18 (28%)

Table S3 (see excel file). Clinical characteristics and morphological features of the presently described individuals with a variant in the MKHK region of *CREBBP* and *EP300*.

The columns of individuals for whom a DNA sample was available are green colored if the diagnosis of the MKHK-subtype was confirmed by episignature testing, and orange colored in case the episignature result did not show any MKHK episignature. [a] Menke et al. 2016[1]; [b] Menke et al. 2018[2]; [c] Angius et al. 2019[3]; [d] Banka et al. 2019.[4] ADHD, attention deficit hyperactivity disorder; ASD, autism spectrum disorder; ASDsf: some features of ASD; ASd: atrial septal defect; BMI, body mass index; DMII, diabetes mellitus type 2; Dpt, diopters; eTSH, elevated thyroid stimulating hormone; GE, gastro-esophageal; GHD, growth hormone deficiency; GI, gastrointestinal; G-tube, gastric tube; ID, intellectual disability; IVC, inferior vena cava; LT, low testosterone level; n.a.; not available; OD, right eye; OFC, occipito-frontal circumference; OS, left eye; PDA, patent ductus arteriosis; PEG, percutaneous gastrostomy; PE-tubes, pressure equalizing tubes; PFO, patent foramen ovale; PVL, periventricular leucomalacia; PVS, Pulmonary valve stenosis; RRTI, recurrent respiratory tract infections; RUTI, recurrent urinary tract infections; SDS, standard deviation score; TSH, thyroid stimulating hormone; TVP, tricuspid valve prolapse; VSD, ventricle septal defect; VUR, vesicoureteral reflux.

Table S4 (see excel file). Genotypes, episignatures and in silico prediction scores of all presently described individuals with a variant in the MKHK region of CREBBP (NM_004380.2) or EP300 (NM_001429.3).

ZZ, Zinc-finger ZZ-type; TAZ2, Zinc-finger TAZ-type; ID4, first α-helix of the fourth intrinsically disordered region. [a]GnomAD: number represents how many times the allele was seen in gnomAD (Genome Aggregation Database) with '.' representing not seen, which is slightly different than '0' (seen but filtered out in the final gnomAD population). CADD, Combined Annotation Dependent Depletion; REVEL, Rare Exome Variant Ensemble Learner; SIFT, Sorting Intolerant From Tolerant; PolyPhen, Polymorphism Phenotyping; MPC, 'Missense badness, PolyPhen-2 and Constraint'.

Table S5. List of identified differentially methylated regions.

Chromosome	Start	End	Width	Number of CpGs	Stouffer	Fisher	Maximum difference	Mean difference	Overlapping gene(s)
**chr17**	40822424	40824241	1818	8	6,98E-77	2,55E-73	0,22913	0,167478	PLEKHH3
**chr6**	26195488	26197606	2119	8	9,12E-36	1,53E-57	-0,25391	-0,10266	AL031777.2,H3C4
**chr6**	27858545	27859478	934	6	1,67E-12	1,07E-27	-0,1179	-0,05217	H3C12
**chr7**	27169136	27171391	2256	21	1,15E-21	3,8E-26	-0,13849	-0,0732	HOXA3,HOXA-AS2,HOXA4,AC004080.6, HOXA-AS3
**chr5**	42756102	42757815	1714	11	1,26E-13	4,4E-24	-0,1223	-0,05729	CCDC152
**chr6**	27782126	27783336	1211	6	1,53E-11	1,74E-19	-0,1646	-0,06806	H2AC14,H2BC14
**chr17**	14205882	14207968	2087	15	1,14E-13	4,14E-19	-0,12428	-0,05383	HS3ST3B1,AC005224.3
**chr5**	1,5E+08	1,5E+08	1504	10	2,12E-11	1,05E-14	0,116828	0,05455	CAMK2A
**chr19**	11517079	11517953	875	7	5,16E-07	1,64E-14	0,142259	0,066341	RGL3
**chr12**	58131345	58133008	1664	9	5,13E-09	5,41E-14	-0,18415	-0,08062	AGAP2,TSPAN31
**chr1**	1,57E+08	1,57E+08	1268	6	5,09E-12	8,5E-14	-0,14	-0,07838	TTC24
**chr6**	26224668	26226256	1589	11	8,64E-12	1,97E-13	-0,1307	-0,0787	H3C6
**chr12**	1641924	1643253	1330	5	2,4E-06	7,74E-12	0,108645	0,056643	WNT5B
**chr9**	98314632	98315816	1185	6	2,28E-06	4,09E-11	-0,109	-0,05646	
**chr1**	27675587	27676652	1066	7	3,33E-12	4,22E-11	-0,09359	-0,06941	SYTL1
**chr10**	70321243	70322874	1632	9	4,52E-06	1,54E-10	-0,11566	-0,05487	TET1
**chr19**	55660514	55661872	1359	6	7,63E-10	2,9E-10	0,156144	0,086835	TNNT1
**chr1**	2,36E+08	2,36E+08	785	6	5,47E-09	2,16E-09	0,109219	0,064921	GNG4
**chr22**	42394048	42395340	1293	11	1,72E-05	4,77E-09	0,115519	0,060151	SEPTIN3,WBP2NL
**chr3**	1,94E+08	1,94E+08	691	7	2,62E-10	9,62E-09	-0,11353	-0,08543	LINC00887
**chr1**	11708564	11709491	928	5	5,48E-07	1,85E-08	0,093936	0,051314	FBXO2
**chr3**	42846594	42847116	523	5	1,09E-06	2,84E-08	-0,11095	-0,05701	AC099329.1,ACKR2
**chr3**	1,41E+08	1,41E+08	544	6	1,94E-08	2,86E-08	-0,0906	-0,06869	ZBTB38
**chr5**	79552470	79553606	1137	5	1,92E-08	4,09E-08	-0,11464	-0,069	
**chr5**	83016630	83017921	1292	11	9,31E-07	4,18E-08	-0,09717	-0,05656	HAPLN1
**chr17**	46719276	46720386	1111	5	6,27E-08	5,18E-08	0,083397	0,055208	AC103702.2
**chr1**	2,35E+08	2,35E+08	645	5	1,48E-06	1,18E-07	-0,08859	-0,05036	
**chr1**	6514605	6516324	1720	5	9,55E-07	2,09E-07	0,096204	0,061211	ESPN
**chr2**	2,43E+08	2,43E+08	481	5	4,77E-06	8,9E-07	0,081801	0,05709	
**chr12**	54412506	54413936	1431	8	6,44E-07	2,07E-06	0,099361	0,065565	AC012531.3,HOXC6,HOXC4,AC012531.2, AC012531.1
**chr10**	21798341	21799395	1055	6	2,59E-06	7,25E-06	-0,09209	-0,06279	
**chr17**	17109640	17110641	1002	11	6,46E-06	7,78E-06	0,097594	0,066414	MPRIP,PLD6,AC055811.2
**chr18**	22907319	22908222	904	5	5,9E-05	8,46E-06	-0,12209	-0,07057	ZNF521
**chr1**	63249197	63249705	509	7	0,002966	9,24E-06	0,113212	0,053852	
**chr1**	86043795	86044230	436	6	2,59E-05	1,07E-05	-0,09664	-0,06059	DDAH1,AC092807.4,AC092807.3
**chr9**	35791475	35792382	908	6	9,94E-05	1,16E-05	-0,10736	-0,05536	NPR2
**chr15**	99408636	99409957	1322	10	2,89E-06	1,37E-05	0,068171	0,053442	IGF1R
**chr19**	36004632	36005395	764	6	4,15E-05	1,84E-05	0,091674	0,060381	
**chr2**	1,64E+08	1,64E+08	716	7	1,17E-06	1,95E-05	-0,09102	-0,06841	
**chr5**	1245669	1246795	1127	6	2,37E-05	2,18E-05	0,094337	0,063162	SLC6A18

**chr17**	78734663	78735596	934	6	3,78E-05	3,15E-05	0,086993	0,054553	RPTOR
**chr1**	27683139	27683912	774	7	6,69E-06	3,29E-05	0,064924	0,052358	MAP3K6
**chr19**	51165207	51165845	639	5	3,59E-05	3,3E-05	0,083174	0,061784	SHANK1
**chr11**	1769152	1770066	915	9	7,68E-05	3,45E-05	0,079182	0,051487	IFITM10,AC068580.4
**chr19**	50249464	50249927	464	5	0,000347	4,02E-05	0,12787	0,07806	TSKS
**chr4**	4763259	4763753	495	7	2,36E-05	6E-05	-0,11503	-0,05714	STX18-AS1
**chr1**	1289806	1291064	1259	8	5,16E-06	6,02E-05	0,097064	0,058265	MXRA8
**chr11**	18477153	18478135	983	7	8,57E-05	6,3E-05	0,12583	0,062944	LDHAL6A
**chr11**	45671208	45672248	1041	5	8,1E-06	6,46E-05	0,087335	0,063194	CHST1
**chr12**	1,31E+08	1,31E+08	759	7	0,000119	8,38E-05	0,109142	0,054137	STX2
**chr9**	1,34E+08	1,34E+08	626	5	4,07E-05	9,53E-05	-0,08873	-0,07043	
**chr19**	49223814	49224454	641	5	3,55E-05	9,9E-05	-0,0969	-0,0565	RASIP1
**chr1**	6187633	6188136	504	5	2,65E-05	0,000131	0,099593	0,068783	CHD5
**chr7**	27127448	27128169	722	6	1,99E-05	0,00015	0,067895	0,050098	
**chr1**	2,44E+08	2,44E+08	1119	5	2,29E-05	0,000164	0,070203	0,052386	ZBTB18
**chr17**	9549496	9550545	1050	8	0,000162	0,000171	-0,09224	-0,05537	USP43,AC118755.1
**chr3**	1,84E+08	1,84E+08	634	7	0,000308	0,000185	0,124694	0,051593	CHRD
**chr10**	75533025	75533431	407	5	0,000418	0,000299	0,102109	0,057383	FUT11
**chr1**	27718221	27718858	638	5	0,000827	0,0003	0,08923	0,050069	
**chr5**	2537210	2537834	625	8	0,000111	0,000382	0,087529	0,053924	
**chr3**	1,6E+08	1,6E+08	480	6	3,71E-05	0,000404	-0,08339	-0,0682	IQCJ-SCHIP1,SCHIP1
**chr4**	1,75E+08	1,75E+08	208	5	0,00032	0,000408	-0,09371	-0,06334	LINC02269,AC106895.2
**chr1**	19971709	19972777	1069	8	5,83E-05	0,00061	-0,10402	-0,06221	NBL1,MICOS10-NBL1
**chr8**	38831681	38832728	1048	6	0,001872	0,001269	-0,07691	-0,05204	HTRA4
**chr11**	834676	835377	702	5	0,004198	0,001496	0,113156	0,061579	CD151
**chr19**	50861774	50862121	348	5	0,000291	0,001703	0,080581	0,060911	NR1H2,NAPSA
**chr6**	1,64E+08	1,64E+08	715	6	0,000407	0,001772	-0,09124	-0,06431	AL078602.1
**chr12**	9217079	9217907	829	11	0,001172	0,002018	-0,10539	-0,06154	LINC00612,A2M-AS1
**chr11**	396686	397486	801	5	0,000753	0,002831	0,095488	0,070649	PKP3
**chr1**	1,12E+08	1,12E+08	474	6	0,001355	0,005106	0,063995	0,052387	C1orf162
**chr1**	47900197	47900327	131	5	0,003204	0,006846	0,079071	0,062555	FOXD2-AS1
**chr22**	38244746	38244902	157	5	0,001669	0,007427	0,070153	0,055552	ANKRD54,EIF3L
**chr2**	20441905	20442210	306	5	0,005672	0,007602	0,078123	0,050274	
**chr10**	1,01E+08	1,01E+08	519	5	0,001764	0,008912	-0,09154	-0,06371	

Start, genomic position of the region's start point; End, genomic position of the region's end point; Width, the width of the region; Stouffer, Stouffer summary transform of the individual CpG false discovery rates (FDRs); Fisher, Fisher combined probability transform of the individual CpG FDRs; Maximum difference: Maximum methylation difference within the DMR; Mean difference: Mean methylation difference across the DMR.

References

1. Menke, L.A., van Belzen, M.J., Alders, M., Cristofoli, F., Ehmke, N., Fergelot, P., Foster, A., Gerkes, E.H., Hoffer, M.J., Horn, D., et al. (2016). CREBBP mutations in individuals without Rubinstein-Taybi syndrome phenotype. Am J Med Genet A *170*, 2681-2693. 10.1002/ajmg.a.37800.
2. Menke, L.A., Gardeitchik, T., Hammond, P., Heimdal, K.R., Houge, G., Hufnagel, S.B., Ji, J., Johansson, S., Kant, S.G., Kinning, E., et al. (2018). Further delineation of an entity caused by CREBBP and EP300 mutations but not resembling Rubinstein-Taybi syndrome. Am J Med Genet A *176*, 862-876. 10.1002/ajmg.a.38626.
3. Angius, A., Uva, P., Oppo, M., Persico, I., Onano, S., Olla, S., Pes, V., Perria, C., Cuccuru, G., Atzeni, R., et al. (2019). Confirmation of a new phenotype in an individual with a variant in the last part of exon 30 of CREBBP. Am J Med Genet A *179*, 634-638. 10.1002/ajmg.a.61052.
4. Banka, S., Sayer, R., Breen, C., Barton, S., Pavaine, J., Sheppard, S.E., Bedoukian, E., Skraban, C., Cuddapah, V.A., and Clayton-Smith, J. (2019). Genotype-phenotype specificity in Menke-Hennekam syndrome caused by missense variants in exon 30 or 31 of CREBBP. Am J Med Genet A *179*, 1058-1062. 10.1002/ajmg.a.61131.
